# Organocatalytic Asymmetric Aldol Reaction of Arylglyoxals and Hydroxyacetone: Enantioselective Synthesis of 2,3-Dihydroxy-1,4-diones

**DOI:** 10.3390/molecules25030648

**Published:** 2020-02-03

**Authors:** Yu-Hao Zhou, Yu-Zu Zhang, Zhu-Lian Wu, Tian Cai, Wei Wen, Qi-Xiang Guo

**Affiliations:** Key Laboratory of Applied Chemistry of Chongqing Municipality, School of Chemistry and Chemical Engineering, Southwest University, Chongqing 400715, China; zyh8182868@email.swu.edu.cn (Y.-H.Z.); zhyzlongzhz@126.com (Y.-Z.Z.); wzl12@swu.edu.cn (Z.-L.W.); caitian@swu.edu.cn (T.C.)

**Keywords:** organocatalysis, aldol reaction, asymmetric synthesis, 2,3-Dihydroxy-1,4-dione, arylglyoxal

## Abstract

A highly efficient quinine-derived primary-amine-catalyzed asymmetric aldol addition of hydroxyacetone to arylglyoxals is described. Structurally diverse *anti*-2,3-dihydroxy-1,4-diones were generated in high yields, with good diastereoselectivities and enantioselectivities.

## 1. Introduction

**Scheme 1:**
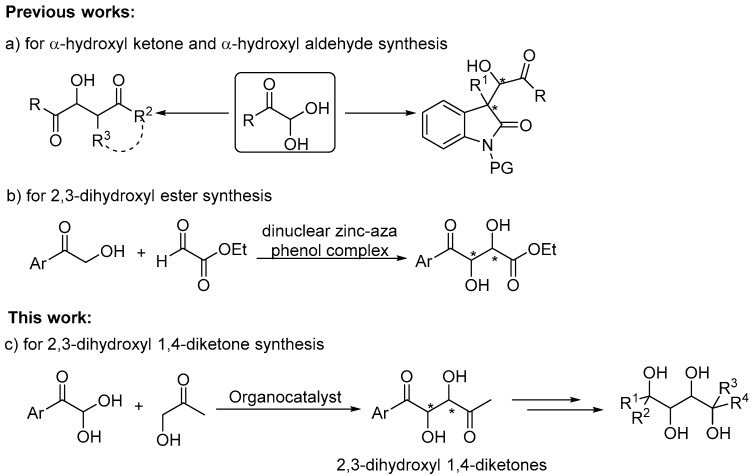
Catalytic asymmetric aldol reaction of glyoxal derivatives.

The enantioselective aldol reaction is among the most important synthetic tools for C–C bond formation in organic synthesis [1,2,3]. Arylglyoxals are endogenous α-oxoaldehydes that have been extensively used as electrophiles in related enantioselective aldol processes to afford synthetically important chiral 2-hydroxy-1,4-dicarbonyl compounds [4,5,6,7,8,9,10,11,12,13,14,15,16,17,18,19]. For example, in 2009, Feng and coworkers developed an asymmetric aldol-type reaction between arylglyoxal derivatives and 2-oxindoles catalyzed by an *N*,*N*′-dioxide-Sc(III) complex [4]. Various optically active 3-substituted oxindoles bearing α-hydroxyl ketone units were produced in excellent yields, with excellent diastereoselectivities and enantioselectivities. Subsequently, Hayashi and coworkers reported a chiral diarylprolinol-catalyzed direct aldol reaction of glyoxal derivatives with aldehydes, affording chiral 2-hydroxy-1,4-dicarbonyl compounds with good experimental outcomes [6]. Zhao and coworkers also reported an aldol reaction between cyclic ketones and arylglyoxals using a cinchona alkaloid-derived thiourea catalyst [10]. Most recently, Wang and coworkers disclosed an aldol reaction of 2-hydroxyacetophenone with ethyl glyoxylate catalyzed by a dinuclear zinc–azaphenol complex [11]. The desired aldol products were obtained with moderate diastereoselectivities. Several other research groups have also reported corresponding catalytic asymmetric aldol or aldol-type reactions, employing phenylglyoxal hydrate in a single example [12,13,14,15,16,17,18,19]. In general, reported examples have mainly focused on the catalytic asymmetric synthesis of α-hydroxyl ketones or 2,3-dihydroxyl esters (Scheme 1a,b), while the catalytic asymmetric construction of 2,3-dihydroxyl carbonyl compounds has yet to be explored. 2,3-Dihydroxyl-1,4-dicarbonyl compounds are known as excellent building blocks for the synthesis of multiple-hydroxyl-containing carbohydrates [20,21]. The direct catalytic asymmetric aldol addition of hydroxyacetone to arylglyoxals is an ideal transformation that can be anticipated to construct such 2,3-dihydroxyl-1,4-carbonyl units (Scheme 1c). Herein, we report a chiral primary amine-catalyzed direct organocatalytic asymmetric aldol reaction of arylglyoxals with hydroxyacetone, which afforded chiral 2,3-dihydroxy-1,4-diones in high yields with good to excellent diastereoselectivities and excellent enantioselectivities.

## 2. Results and Discussion

### 2.1. Optimization of Reaction Conditions

Phenylglyoxal monohydrate (**1a**) and hydroxyacetone (**2a**) were selected as model reactants for initial tests, with various natural amino acids, including l-Proline, l-Serine, and l-Threonine, screened as catalysts [22,23] and *N*,*N*-dimethyl formamide (DMF) as solvent. Unfortunately, desired product **4a** was not obtained (Table 1, entries 1–3). Chiral pyrrolidine derivatives **3a** and **3b** were also ineffective in this reaction (entries 4 and 5). Next, chiral *trans*-*N*,*N*-dialkyl diaminocyclohexanes **3c**–**3e** were used as catalysts to promote this reaction [24], giving desired product **4a** in good yields, with good diastereoselectivities and excellent enantioselectivities (entries 6–8). Catalyst **3f**, derived from (1*S*,2*S*)-1,2-diphenylethane-1,2-diamine, was also tested, but generated product **4a** with poor diastereoselectivity and moderate enantioselectivity (entry 9). The stereoselectivity of **4a** was slightly enhanced when quinine-derived primary amine **3g** was used as catalyst (84% yield, 94% ee, 89:11 dr; entry 10). Based on these results, chiral primary amine **3g** was selected as the best catalyst for further optimization of the reaction conditions. Next, the effect of Brønsted acid additives was studied [25]. When additive 3,5-dinitrobenzoic acid (DNBA) was replaced by a weaker acid, *p*-nitrobenzoic acid, the reaction was slower and the yield, diastereoselectivity, and enantioselectivity of **4a** were decreased slightly (entry 11). Similar results were observed when DNBA was replaced with tosic acid (entry 12). These results indicated that DNBA was the most suitable acid additive for this reaction. Decreasing the reaction temperature clearly enhanced the stereoselectivity. For example, when the reaction was conducted at 0 °C, **4a** was obtained with 96% ee and 93:7 dr (entry 13). With the aim to further enhance the experimental outcome of **4a** by introducing another hydrogen donor group, catalyst **3h** was prepared. However, both the yield and stereoselectivity of **4a** were decreased slightly in the reaction promoted by **3h** (entry 14). An investigation of the catalyst loading indicated that 10 mol% of **3g** was sufficiently effective for this reaction (entry 15). Further decreasing the catalyst loading greatly diminished the reaction outcome (entry 16). Various solvents were examined using catalyst **3g**, with the results indicating that CHCl_3_ was the best solvent in terms of yield, diastereoselectivity, and enantioselectivity. Based on these results, the reaction conditions depicted in entry 15 were selected as optimal for further substrate scope investigations.

### 2.2. Substrate Scope Study

**Figure 1:**
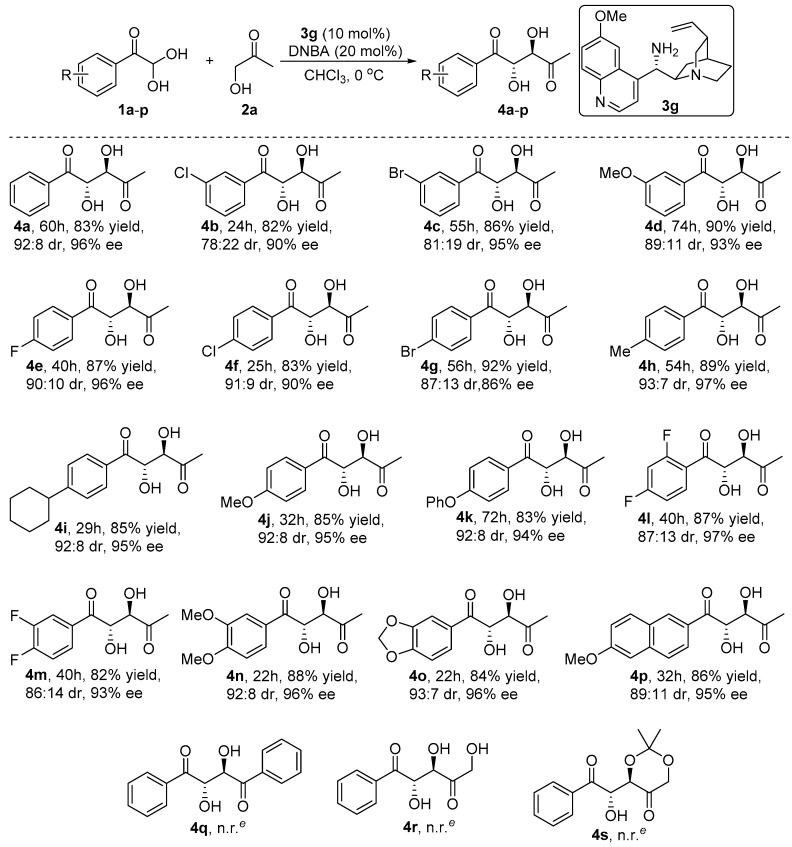
Substrate scope study *^a^*^–*d*^. *^a^* All reactions were performed with **1** (0.2 mmol), **2a** (1.0 mmol), DNBA (0.04 mmol), and **3g** (0.02 mmol) in CHCl_3_ (2.0 mL) at 0 °C. *^b^* Isolated yield. *^c^* dr determined by ^1^H-NMR. *^d^* ee of *anti*-diastereomer determined by chiral HPLC. *^e^* n.r. = no reaction.

Under the optimized reaction conditions, *anti*-selective aldol reactions of hydroxyacetone **2a** with various phenylglyoxals **1b**–**1p** were examined (Figure 1). In addition to phenylglyoxal monohydrate **1a**, substituted arylglyoxal monohydrates were found to be good reaction partners in this transformation. The electronic properties and positions of substituents on the phenyl ring had almost no influence on the reactivity and stereoselectivity. For example, arylglyoxals bearing 3-Cl-, Br-, or MeO-substituted phenyl groups reacted with hydroxyacetone smoothly to give the corresponding 2,3-dihydroxy-1,4-diketone products **4b**–**4m** in high yields (82–92%) with high enantioselectivities (86–93% ee). For arylglyoxals containing *para*-substituted phenyl groups, both electron-deficient and electron-rich phenyl-substituted substrates afforded products with excellent yields, diastereoselectivities, and enantioselectivities (Figure 1, **4e**–**4k**). Arylglyoxals bearing disubstituted phenyls were also good reaction partners, affording products **4l**–**4o** with excellent experimental outcomes. 6-Methoxy-2-naphthyl-substituted arylglyoxal reacted with **2a** to giving product **4p** in excellent yield (86%), with high diastereoselectivity (89:11 dr) and excellent enantioselectivity (95% ee). We also explored other hydroxy ketones, including α-hydroxyacetophenone, 1,3-dihydroxyacetone, and ketal-protected 1,3-dihydroxyacetone as donors, which reacted with **1a** to obtain **4q**–**4s**, but failed. Although all arylglyoxals tested in this reaction gave excellent experimental outcomes, the substrate scope of this reaction could not be expanded further owing to the limited available of arylglyoxal and α-hydroxyketone starting materials.

### 2.3. Scale-Up Experiment and Crystal Structure of Compound **4j**

**Scheme 2:**
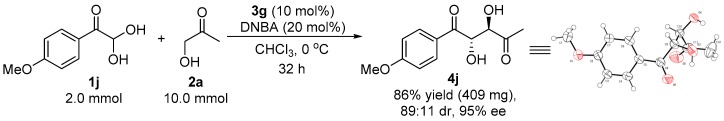
Scaled-up 2-mmol reaction.

Notably, this reaction was successfully conducted on a 2-mmol scale (Scheme 2), with product **4j** obtained in 86% yield with 95% ee and 89:11 dr. The relative and absolute configurations of **4j** (2*S*,3*R*) were determined by X-ray crystallography (see the Supplementary Materials) [26]. The stereochemistry of the other aldol products was assigned by comparison with **4j**.

### 2.4. Plausible Reaction Mechanism and Transition States

**Scheme 3:**
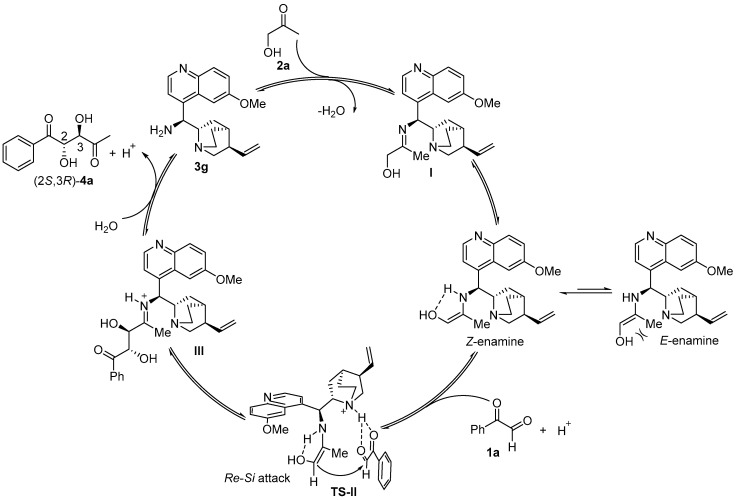
Plausible reaction mechanism.

A proposed catalytic cycle and transition state model [27] are shown in Scheme 3. The condensation of catalyst **3g** with hydroxyacetone **2a** affords imine intermediate **I**. Intermediate **I** then isomerizes to form a (*Z*)-enamine, with an intramolecular N–H^...^O hydrogen bond assumed to play a critical role in stabilizing the *Z*-enamine [23]. Meanwhile, the arylglyoxal is activated by hydrogen-bond formation with the protonated nitrogen atom of the quinuclidine. In the proposed transition state, the *Re*-face of the enamine attacks the *Si*-face of the arylglyoxal to give intermediate **III**. Finally, intermediate **III** undergoes hydrolysis to afford **4a** with a 2*S*,3*R*-conformation. The Brønsted acid additive facilitates the enamine catalytic cycle.

## 3. Materials and Methods

### 3.1. General Information

Unless otherwise noted, commercial reagents were used as received. All reactions were monitored by TLC with silica gel coated plates. ^1^H-NMR (600 MHz) and ^13^C-NMR (150 MHz) spectra were recorded on Bruker Avance 600 MHz spectrometer. Chemical shifts (δ) are reported in ppm from tetramethylsilane (TMS) with the solvent resonance as the internal standard. Proton gsignal multiplicities are given as s (singlet), d (doublet), t (triplet), q (quartet), m (multiplet), br (broad) or a combination of them. *J*-values are in Hz. HRMS (ESI-Q-TOF) spectra were recorded on Bruker Impact-II. Enantiomer ratios were determined by HPLC with chiral columns (Chiralpak AD-H, IC-H, IA-H, OD-H, OJ-H columns were purchased from Daicel Chemical Industries, LTD.). Optical rotations were determined at λ = 589 nm (sodium D line) by using a Rudolph-API automatic polarimeter (Hackettstown, NJ, USA).

### 3.2. General Procedure for the Synthesis of 2,3-Dihydroxy-1,4-dione Diketone Products **4a**–**4p**

A dry tube was charged with **1** (0.2 mmol), **2** (1 mmol), catalyst **3g** (0.02 mmol) and DNBA (0.04 mmol). After addition of CHCl_3_ (2.0 mL), the mixture was effectively stirred at 0 °C and monitored by TLC. After the complete consumption of compound **1,** the mixture was concentrated in vacuo and purified by flash chromatography on silica gel (PE:Et_2_O = 1:1.5) to afford diastereomeric mixtures of **4a**–**4p** (yield: 82–92%), see Supplementary Materials.

*(2S,3R)-2,3-dihydroxy-1-phenylpentane-1,4-dione*. Product **4a** was obtained as a white solid in 83% yield after flash chromatography and the enantiometric excess was determined to be 96% by HPLC analysis on Daicel Chirapak AD-H column (hexane/isopropanol = 80/20, flow rate 1.0 mL/min, T = 30 °C, *t_R_* (major) 9.298 min, *t_R_* (minor) 10.169 min), m.p. 53.3−55.3 °C; [α${]}_{D}^{25}$ = −31.03 (c = 0.145, CH_2_Cl_2_). The diastereomeric ratio was determined to be 92: 8 by ^1^H-NMR on Bruker Avance 600 spectrometer; ^1^H-NMR (600 MHz, CDCl_3_) δ 7.97 (d, *J* = 7.6 Hz, 2H), 7.64 (t, *J* = 7.4 Hz, 1H), 7.52 (t, *J* = 7.7 Hz, 2H), 5.28 (s, 1H), 4.47 (s, 1H), 4.05 (d, *J* = 5.7 Hz, 1H), 3.94 (d, *J* = 5.8 Hz, 1H), 2.13 (s, 3H); ^13^C-NMR (151 MHz, CDCl_3_) δ 207.38, 199.04, 134.43, 129.09, 129.00, 79.66, 75.86, 27.43; HRMS(ESI): calcd. for C_11_H_12_NaO_4_(M^+^ + Na): 231.0628, found 231.0628.

*(2S,3R)-1-(3-chlorophenyl)-2,3-dihydroxypentane-1,4-dione*. Product **4b** was obtained as a white solid in 82% yield after flash chromatography and the enantiometric excess was determined to be 90% by HPLC analysis on Daicel Chirapak IC-H column (hexane/isopropanol = 85/15, flow rate 1.0 mL/min, T = 30 °C, *t_R_* (major) 9.905 min, *t_R_* (minor) 10.732 min), m.p. 59.1–61.9 °C; [α${]}_{D}^{25}$ = −22.78 (c = 0.237, CH_2_Cl_2_). The diastereomeric ratio was determined to be 78: 22 by ^1^H-NMR on Bruker Avance 600 spectrometer; ^1^H-NMR (600 MHz, CDCl_3_) δ 7.95 (s, 1H), 7.84 (d, *J* = 7.8 Hz, 1H), 7.64–7.55 (m, 1H), 7.45 (t, *J* = 7.9 Hz, 1H), 5.17 (d, *J* = 4.1 Hz, 1H), 4.41 (d, *J* = 4.1 Hz, 1H), 3.91 (d, *J* = 58.8 Hz, 2H), 2.21 (s, 3H); ^13^C-NMR (151 MHz, CDCl_3_) δ 207.51, 197.96, 136.07, 135.29, 134.07, 130.16, 128.90, 127.01, 79.19, 75.53, 27.31. HRMS(ESI): calcd. for C_11_H_11_ClNaO_4_(M^+^ + Na): 265.0238, found 265.0234.

*(2S,3R)-1-(3-bromophenyl)-2,3-dihydroxypentane-1,4-dione*. Product **4c** was obtained as a white solid in 86% yield after flash chromatography and the enantiometric excess was determined to be 95% by HPLC analysis on Daicel Chirapak AD-H column (hexane/isopropanol = 70/30, flow rate 1.0 mL/min, T = 30 °C, *t_R_* (major) 6.140 min, *t_R_* (minor) 4.920min), m.p. 66.5–68.1 °C; [α${]}_{D}^{25}$ = −14.91 (c = 0.248, CH_2_Cl_2_). The diastereomeric ratio was determined to be 81: 19 by ^1^H-NMR on Bruker Avance 600 spectrometer; ^1^H-NMR (600 MHz, CDCl_3_) δ 8.11 (s, 1H), 7.89 (d, *J* = 7.7 Hz, 1H), 7.76 (d, *J* = 7.8 Hz, 1H), 7.40 (t, *J* = 7.9 Hz, 1H), 5.16 (s, 1H), 4.41 (s, 1H), 3.86 (d, *J* = 61.5 Hz, 2H), 2.21 (s, 3H); ^13^C-NMR (151 MHz, CDCl_3_) δ 207.43, 197.88, 137.05, 136.22, 131.83, 130.40, 127.47, 123.22, 79.18, 75.42, 27.36. HRMS(ESI): calcd. For C_11_H_11_BrNaO_4_(M^+^ + Na): 308.9733, found 308.9735.

*(2S,3R)-2,3-dihydroxy-1-(3-methoxyphenyl)pentane-1,4-dione*. Product **4d** was obtained as an oil in 90% yield after flash chromatography and the enantiometric excess was determined to be 93% by HPLC analysis on Daicel Chirapak AD-H column (hexane/isopropanol = 70/30, flow rate 1.0 mL/min, T = 30 °C, *t_R_* (major) 7.784 min, *t_R_* (minor) 9.664 min), [α${]}_{D}^{25}$ = −4.73 (c = 0.169, CH_2_Cl_2_). The diastereomeric ratio was determined to be 89: 11 by ^1^H-NMR on Bruker Avance 600 spectrometer; ^1^H-NMR (600 MHz, CDCl_3_) δ 7.54 (d, *J* = 7.6 Hz, 1H), 7.49 (s, 1H), 7.42 (t, *J* = 7.9 Hz, 1H), 7.18 (dd, *J* = 8.2, 2.3 Hz, 1H), 5.25 (d, *J* = 2.6 Hz, 1H), 4.49 (d, *J* = 3.0 Hz, 1H), 3.87 (s, 3H), 2.13 (s, 3H); ^13^C-NMR (151 MHz, CDCl_3_) δ 207.26, 198.68, 160.05, 135.52, 129.96, 121.32, 120.77, 113.18, 79.64, 75.86, 55.53, 27.31. HRMS(ESI): calcd. for C_12_H_14_NaO_5_(M^+^ + Na): 261.0733, found 261.0730.

*(2S,3R)-1-(4-fluorophenyl)-2,3-dihydroxypentane-1,4-dione*. Product **4e** was obtained as a white solid in 87% yield after flash chromatography and the enantiometric excess was determined to be 96% by HPLC analysis on Daicel Chirapak AD-H column (hexane/isopropanol = 80/20, flow rate 1.0 mL/min, T = 30 °C, *t_R_* (major) 8.384 min, *t_R_* (minor) 8.987 min), m.p. 67.2–69.3 °C; [α${]}_{D}^{25}$ = −31.62 (c = 0.117, CH_2_Cl_2_) The diastereomeric ratio was determined to be 90: 10 by ^1^H-NMR on Bruker Avance 600 spectrometer; ^1^H-NMR (600 MHz, CDCl_3_) δ 8.03 (dd, *J* = 7.9, 5.7 Hz, 2H), 7.19 (t, *J* = 8.4 Hz, 2H), 5.19 (s, 1H), 4.41 (s, 1H), 3.99 (d, *J* = 6.6 Hz, 1H), 3.88 (d, *J* = 6.6 Hz, 1H), 2.19 (s, 3H); ^13^C-NMR (151 MHz, CDCl_3_) δ 207.61, 197.59, 167.39, 165.68, 131.98, 131.92, 116.40, 116.25, 79.43, 75.49, 27.50. HRMS(ESI): calcd. for C_11_H_11_NaO_4_(M^+^ + Na): 249.0534, found: 249.0534.

*(2S,3R)-1-(4-chlorophenyl)-2,3-dihydroxypentane-1,4-dione*. Product **4f** was obtained as a white solid in 83% yield after flash chromatography and the enantiometric excess was determined to be 90% by HPLC analysis on Daicel Chirapak IA-H column (hexane/isopropanol = 80/20, flow rate 1.0 mL/min, T = 30 °C, *t_R_* (major) 9.256 min, *t_R_* (minor) 9.740 min), m.p. 81.0–83.1 °C; [α${]}_{D}^{25}$ = −10.97 (c = 0.164, CH_2_Cl_2_). The diastereomeric ratio was determined to be 91: 9 by ^1^H-NMR on Bruker Avance 600 spectrometer; ^1^H-NMR (600 MHz, CDCl_3_) δ 7.93 (dd, *J* = 5.4, 3.1 Hz, 2H), 7.49 (dd, *J* = 5.4, 3.1 Hz, 2H), 5.16 (s, 1H), 4.38 (s, 1H), 3.98–3.71 (m, 2H), 2.20 (s, 3H); ^13^C-NMR (151 MHz, CDCl_3_) δ 207.40, 197.92, 140.94, 132.73, 130.37, 129.28, 79.22, 75.31, 27.37. HRMS(ESI): calcd. For C_11_H_11_ClNaO_4_(M^+^ + Na): 265.0238, found 265.0232.

*(2S,3R)-1-(4-bromophenyl)-2,3-dihydroxypentane-1,4-dione*. Product **4g** was obtained as a white solid in 92% yield after flash chromatography and the enantiometric excess was determined to be 86% by HPLC analysis on Daicel Chirapak IA-H column (hexane/isopropanol = 90/10, flow rate 1.0 mL/min, T = 30 °C, *t_R_* (major) 18.602 min, *t_R_* (minor) 20.004 min), m.p. 98.2–100.2 °C; [α${]}_{D}^{25}$ = −26.82 (c = 0.205, CH_2_Cl_2_). The diastereomeric ratio was determined to be 87: 13 by ^1^H-NMR on Bruker Avance 600 spectrometer; ^1^H-NMR (600 MHz, CDCl_3_) δ 7.84 (dd, *J* = 8.4, 2.1 Hz, 2H), 7.65 (dd, *J* = 8.1, 2.8 Hz, 2H), 5.18 (s, 1H), 4.40 (s, 1H), 4.03 (d, *J* = 26.9 Hz, 1H), 3.93 (d, *J* = 33.1 Hz, 1H), 2.18 (s,3H); ^13^C-NMR (151 MHz, CDCl_3_) δ 207.66, 198.27, 133.29, 132.39, 130.53, 129.78, 79.37, 75.53, 27.48. HRMS(ESI): calcd for C_11_H_11_BrNaO_4_(M^+^ + Na): 308.9733, found 308.9732.

*(2S,3R)-2,3-dihydroxy-1-p-tolylpentane-1,4-dione*. Product **4h** was obtained as a white solid in 89% yield after flash chromatography and the enantiometric excess was determined to be 97% by HPLC analysis on Daicel Chirapak AD-H column (hexane/isopropanol = 70/30, flow rate 1.0 mL/min, T = 30 °C, *t_R_* (major) 7.000 min, *t_R_* (minor) 7.893 min), m.p. 86.1–89.7 °C; [α${]}_{D}^{25}$ = −2.49 (c = 0.281, CH_2_Cl_2_). The diastereomeric ratio was determined to be 93: 7 by ^1^H-NMR on Bruker Avance 600 spectrometer; ^1^H-NMR (600 MHz, CDCl_3_) δ 7.89 (d, *J* = 8.1 Hz, 2H), 7.33 (d, *J* = 7.9 Hz, 2H), 5.24 (d, *J* = 3.5 Hz, 1H), 4.46 (d, *J* = 3.5 Hz, 1H), 3.89 (ddd, *J* = 95.5, 59.2, 31.9 Hz, 2H), 2.44 (s, 3H), 2.13 (d, *J* = 11.8 Hz, 3H); ^13^C-NMR (151 MHz, CDCl_3_) δ 207.03, 198.36, 145.63, 131.62, 129.69, 129.02, 79.64, 75.60, 27.35, 21.78. HRMS(ESI): calcd. for C_12_H_14_NaO_4_(M^+^ + Na): 245.0784, found 245.0787.

*(2S,3R)-1-(4-cyclohexylphenyl)-2,3-dihydroxypentane-1,4-dione*. Product **4i** was obtained as a white solid in 85% yield after flash chromatography and the enantiometric excess was determined to be 95% by HPLC analysis on Daicel Chirapak AD-H column (hexane/isopropanol = 70/30, flow rate 1.0 mL/min, T = 30 °C, *t_R_* (major) 7.562 min, *t_R_* (minor) 9.270 min), m.p. 66.1–68.1 °C; [α${]}_{D}^{25}$ = −11.46 (c = 0.218, CH_2_Cl_2_). The diastereomeric ratio was determined to be 92: 8 by ^1^H-NMR on Bruker Avance 600 spectrometer; ^1^H-NMR (600 MHz, CDCl_3_) δ 7.92 (d, *J* = 8.0 Hz, 2H), 7.35 (d, *J* = 8.1 Hz, 2H), 5.24 (d, *J* = 2.8 Hz, 1H), 4.47 (s, 1H), 3.85 (d, *J* = 72.9 Hz, 2H), 2.60 (d, *J* = 8.4 Hz, 1H), 2.12 (s, 3H), 1.88 (s, 4H), 1.78 (d, *J* = 12.8 Hz, 1H), 1.43 (d, *J* = 9.1 Hz, 5H); ^13^C-NMR (151 MHz, CDCl_3_) δ 206.89, 198.39, 155.49, 131.83, 129.17, 127.57, 79.67, 75.68, 44.86. 34.03, 27.39, 26.69, 26.01. HRMS(ESI): calcd. for C_11_H_22_NaO_4_(M^+^ + Na): 313.1410, found 313.1413.

*(2S,3R)-2,3-dihydroxy-1-(4-methoxyphenyl)pentane-1,4-dione*. Product **4j** was obtained as an oil in 85% yield after flash chromatography and the enantiometric excess was determined to be 95% by HPLC analysis on Daicel Chirapak AD-H column (hexane/isopropanol = 70/30, flow rate 1.0 mL/min, T = 30 °C, *t_R_* (major) 8.752 min, *t_R_* (minor) 9.923 min). [α${]}_{D}^{25}$ = −16.04 (c = 0.187, CH_2_Cl_2_). The diastereomeric ratio was determined to be 92: 8 by ^1^H-NMR on Bruker Avance 600 spectrometer; ^1^H-NMR (600 MHz, CDCl_3_) δ 7.99 (d, *J* = 8.8 Hz, 2H), 6.99 (d, *J* = 8.8 Hz, 2H), 5.22 (dd, *J* = 5.7, 4.2 Hz, 1H), 4.45 (dd, *J* = 6.1, 4.1 Hz, 1H), 4.05 (d, *J* = 6.0 Hz, 1H), 3.95 (d, *J* = 6.8 Hz, 1H), 3.89 (s, 3H), 2.13 (s, 3H); ^13^C-NMR (151 MHz, CDCl_3_) δ 207.37, 197.17, 164.74, 131.54, 127.06, 114.38, 79.82, 75.48, 55.72, 27.52. HRMS(ESI): calcd for C_12_H_14_NaO_5_(M^+^ + Na): 261.0736, found 261.0736.

*(2S,3R)-2,3-dihydroxy-1-(4-phenoxyphenyl)pentane-1,4-dione*. Product **4k** was obtained as an oil in 83% yield after flash chromatography and the enantiometric excess was determined to be 94% by HPLC analysis on Daicel Chirapak IC-H column (hexane/isopropanol = 70/30, flow rate 1.0 mL/min, T = 30 °C, *t_R_* (major) 7.932 min, *t_R_* (minor) 8.424 min), [α${]}_{D}^{25}$ = −2.08 (c = 0.048, CH_2_Cl_2_). The diastereomeric ratio was determined to be 92: 8 by ^1^H-NMR on Bruker Avance 600 spectrometer; ^1^H-NMR (600 MHz, CDCl_3_) δ 7.97 (d, *J* = 8.8 Hz, 2H), 7.41 (t, *J* = 7.9 Hz, 2H), 7.22 (s, 1H), 7.08 (d, *J* = 7.7 Hz, 2H), 7.02 (d, *J* = 8.8 Hz, 2H), 5.19 (d, *J* = 3.4 Hz, 1H), 4.44 (d, *J* = 3.7 Hz, 1H), 2.16 (s, 3H); ^13^C-NMR (151 MHz, CDCl_3_) δ 207.38, 197.23, 163.22, 154.95, 131.42, 130.17, 128.51, 125.07, 120.47, 117.34, 79.55, 75.38, 27.41. HRMS(ESI): calcd. for C_11_H_16_NaO_5_(M^+^ + Na): 323.0890, found 323.0886.

*(2S,3R)-1-(2,4-difluorophenyl)-2,3-dihydroxypentane-1,4-dione*. Product **4l** was obtained as an oil in 87% yield after flash chromatography and the enantiometric excess was determined to be 97% by HPLC analysis on Daicel Chirapak AD-H column (hexane/isopropanol = 80/20, flow rate 1.0 mL/min, T = 30 °C, *t_R_* (major) 8.257 min, *t_R_* (minor) 9.181 min), [α${]}_{D}^{25}$ = −16.08 (c = 0.143, CH_2_Cl_2_) The diastereomeric ratio was determined to be 87: 13 by ^1^H-NMR on Bruker Avance 600 spectrometer; ^1^H-NMR (600 MHz, CDCl_3_) δ 7.98 (dd, *J* = 15.0, 8.3 Hz, 1H), 7.10 – 7.00 (m, 1H), 6.95–6.87 (m, 1H), 5.10 (s, 1H), 4.35 (s, 1H), 4.01 (d, *J* = 5.5 Hz, 1H), 3.67 (d, *J* = 5.8 Hz, 1H), 2.24 (s, 3H); ^13^C-NMR (151 MHz, CDCl_3_) δ 207.97, 195.81, 195.79, 167.56, 167.48, 165.85, 165.76, 163.40, 163.32, 161.69, 161.61, 133.31, 133.28, 133.24, 133.21, 113.13, 113.11, 112.98, 112.96, 105.25, 105.08, 104.90, 79.43, 78.08, 78.03, 27.27. HRMS(ESI): calcd. for C_11_H_10_F_2_NaO_4_(M^+^ + Na): 267.0439, found 267.0446.

*(2S,3R)-1-(3,4-difluorophenyl)-2,3-dihydroxypentane-1,4-dione*. Product **4m** was obtained as a white solid in 82% yield after flash chromatography and the enantiometric excess was determined to be 93% by HPLC analysis on Daicel Chirapak OJ-H column (hexane/isopropanol = 80/20, flow rate 1.0 mL/min, T = 30 °C, *t_R_* (major) 8.205 min, *t_R_* (minor) 8.988 min), m.p. 68.6–70.4 °C; [α${]}_{D}^{25}$ = −13.79 (c = 0.087, CH_2_Cl_2_) The diastereomeric ratio was determined to be 86: 14 by ^1^H-NMR on Bruker Avance 600 spectrometer; ^1^H-NMR (600 MHz, CDCl_3_) δ 7.86 (t, *J* = 8.3 Hz, 1H), 7.82–7.75 (m, 1H), 7.30 (dd, *J* = 17.1, 8.6 Hz, 1H), 5.11 (s, 1H), 4.37 (s, 1H), 3.91 (s, 1H), 3.83 (s, 1H), 2.25 (s, 3H); ^13^C-NMR (151 MHz, CDCl_3_) δ 207.73, 196.96, 155.33, 155.25, 153.61, 153.53, 151.54, 151.45, 149.87, 149.78, 131.70, 126.50, 126.47, 126.45, 126.42, 118.72, 118.60, 118.05, 117.93, 79.15, 75.31, 27.50. HRMS(ESI): calcd. for C_11_H_10_F_2_NaO_4_(M^+^ + Na): 267.0439, found: 267.0441.

*(2S,3R)-1-(3,4-dimethoxyphenyl)-2,3-dihydroxypentane-1,4-dione*. Product **4n** was obtained as a white solid in 88% yield after flash chromatography and the enantiometric excess was determined to be 97% by HPLC analysis on Daicel Chirapak AD-H column (hexane/isopropanol = 80/20 min, flow rate 1.0 mL/min, T = 30 °C, *t_R_* (major) 14.002, *t_R_* (minor) 15.915 min), m.p. 73.0–74.6 °C; [α${]}_{D}^{25}$ = −14.41 (c = 0.215, CH_2_Cl_2_). The diastereomeric ratio was determined to be 92: 8 by ^1^H-NMR on Bruker Avance 600 spectrometer; ^1^H-NMR (600 MHz, CDCl_3_) δ 7.64 (d, *J* = 8.4 Hz, 1H), 7.55 (s, 1H), 6.94 (d, *J* = 8.4 Hz, 1H), 5.23 (dd, *J* = 5.6, 4.3 Hz, 1H), 4.50–4.44 (m, 1H), 4.03 (dd, *J* = 10.9, 6.0 Hz, 1H), 3.97 (s, 3H), 3.95 (s, 3H), 3.94–3.89 (m, 1H), 2.15 (s, 3H); ^13^C-NMR (151 MHz, CDCl_3_) δ 207.43, 197.19, 154.69, 149.60, 127.17, 124.17, 111.06, 110.48, 79.95, 75.40, 56.30, 56.21, 27.55. HRMS(ESI): calcd for C_13_H_16_NaO_6_(M^+^ + Na): 291.0839, found 291.0844.

*(2S,3R)-1-(benzo[d][1,3]dioxol-5-yl)-2,3-dihydroxypentane-1,4-dione*. Product **4o** was obtained as a white solid in 84% yield after flash chromatography and the enantiometric excess was determined to be 96% by HPLC analysis on Daicel Chirapak AD-H column (hexane/isopropanol = 80/20, flow rate 1.0 mL/min, T = 30 °C, *t_R_* (major) 21.336 min, *t_R_* (minor) 16.964 min), m.p. 73.9–75.2 °C; [α${]}_{D}^{25}$ = −9.55 (c = 0.314, CH_2_Cl_2_). The diastereomeric ratio was determined to be 93: 7 by ^1^H-NMR on Bruker Avance 600 spectrometer; ^1^H-NMR (600 MHz, CDCl_3_) δ 7.61 (d, *J* = 8.2 Hz, 1H), 7.47 (s, 1H), 6.90 (d, *J* = 8.2 Hz, 1H), 6.08 (s, 2H), 5.16 (dd, *J* = 6.0, 4.3 Hz, 1H), 4.44 (dd, *J* = 6.7, 4.2 Hz, 1H), 4.00 (d, *J* = 6.2 Hz, 1H), 3.92 (d, *J* = 6.6 Hz, 1H), 2.15 (s, 3H); ^13^C-NMR (151 MHz, CDCl_3_) δ 207.41, 196.86, 153.12, 148.65, 128.85, 125.88, 108.66, 108.42, 102.33, 79.77, 75.46, 27.55. HRMS(ESI): calcd for C_12_H_12_NaO_6_(M^+^ + Na): 275.0526, found 275.0528.

*(2S,3R)-2,3-dihydroxy-1-(6-methoxynaphthalen-2-yl)pentane-1,4-dione*. Product **4p** was obtained as a white solid in 89% yield after flash chromatography and the enantiometric excess was determined to be 97% by HPLC analysis on Daicel Chirapak IA-H column (hexane/isopropanol = 80/20, flow rate 1.0 mL/min, T = 30 °C, *t_R_* (major) 15.424 min, *t_R_* (minor) 18.251 min), m.p. 131.2–132.3 °C; [α${]}_{D}^{25}$ = −48.63 (c = 0.183, CH_2_Cl_2_). The diastereomeric ratio was determined to be 91: 9 by ^1^H-NMR on Bruker Avance 600 spectrometer; ^1^H-NMR (600 MHz, CDCl_3_) δ 8.45 (s, 1H), 7.99 (d, *J* = 8.6 Hz, 1H), 7.87 (d, *J* = 9.0 Hz, 1H), 7.81 (d, *J* = 8.6 Hz, 1H), 7.23 (dd, *J* = 8.9, 2.2 Hz, 1H), 7.16 (s, 1H), 5.48–5.33 (m, 1H), 4.55 (dd, *J* = 6.4, 4.0 Hz, 1H), 4.06 (d, *J* = 6.2 Hz, 1H), 4.01–3.86 (m, 4H), 2.13 (s, 3H); ^13^C-NMR (151 MHz, CDCl_3_) δ 207.23, 198.41, 160.63, 138.17, 131.56, 131.11, 129.45, 127.85, 127.77, 124.87, 120.35, 106.08, 79.93, 75.80, 55.63, 27.56. HRMS(ESI): calcd for C_16_H_16_NaO_5_(M^+^ + Na): 311.0890, found 311.0877.

## 4. Conclusions

In summary, we have developed a highly stereoselective *anti*-aldol reaction of arylglyoxal monohydrates with hydroxyacetone catalyzed by quinine-derived primary amine **3g**. The desired 2,3-dihydroxy-1,4-dione products were obtained in high yields (up to 92%), with excellent enantioselectivities (up to 97% ee) and diastereoselectivities (up to 93:7 dr).

## Figures and Tables

**Table 1:**
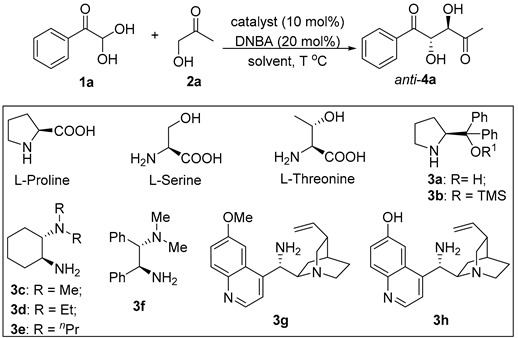
Optimization of reaction conditions *^a^*.

Entry	Catalyst	Solvent	Time (h)	Yield *^b^* (%)	dr ^c^(*anti:syn*)	ee *^c^* (%)
1 ^d,e^	L-Pro	DMF	24	n.r.	n.d.	n.d.
2 ^d,e^	L-Ser	DMF	60	n.r.	n.d.	n.d.
3 ^d,e^	L-Thr	DMF	60	n.r.	n.d.	n.d.
4	**3a**	CHCl_3_	143	trace	n.d.	n.d.
5	**3b**	CHCl_3_	234	trace	n.d.	n.d.
6	**3c**	CHCl_3_	16	49	80:20	94
7	**3d**	CHCl_3_	13	85	80:20	94
8	**3e**	CHCl_3_	12	82	86:14	92
9	**3f**	CHCl_3_	80	62	57:43	−57
10 *^e^*	**3g**	CHCl_3_	13	84	89:11	94
11 *^e,f^*	**3g**	CHCl_3_	84	74	75:25	92
12 *^e,g^*	**3g**	CHCl_3_	15	75	86:14	90
13 *^e^*^,*h*^	**3g**	CHCl_3_	46	89	93:7	96
14 *^e^*^,*h*^	**3h**	CHCl_3_	60	85	87:13	89
15 *^h^*	**3g**	CHCl_3_	60	83	93:7	96
16 *^h,i^*	**3g**	CHCl_3_	108	59	93:7	96
17 *^h^*	**3g**	CH_2_Cl_2_	60	17	87:13	95
18 *^h^*	**3g**	DCE	60	30	87:13	94
19 *^h^*	**3g**	PhCH_3_	60	48	87:13	88

*^a^* Unless otherwise noted, reactions were performed with **1a** (0.2 mmol), **2a** (1.0 mmol), DNBA (0.02 mmol), and catalyst (0.04 mmol) in the specified solvent (2.0 mL) at 30 °C. *^b^* Isolated yield. *^c^* Determined by chiral HPLC. *^d^* Reaction performed in the absence of DNBA. *^e^* 20 mol% of catalyst used. *^f^ p*-NO_2_C_6_H_4_CO_2_H (0.02 mmol) used instead of DNBA. *^g^* TsOH^•^H_2_O (0.02 mmol) used instead of DNBA. *^h^* Reaction performed at 0 °C. *^i^* 5 mol% of **3g** used. DNBA = 3,5-dinitrobenzoic acid; DCE = 1,2-dichloroethene; n.r. = no reaction; n.d. = not determined.

## Supplementary Materials

Supporting information including the spectrums of ^1^H, ^13^C-NMR and HPLC are available online.
