# Supplementary material for: Organocatalytic Asymmetric Aldol Reaction of Arylglyoxals and Hydroxyacetone: Enantioselective Synthesis of 2,3-Dihydroxy-1,4-diones
Source: Molecules. 2020 Feb 3;25(3):648. doi: 10.3390/molecules25030648 (PMC7037326; doi:10.3390/molecules25030648)
Supplement: Supplementary file 1 [file molecules-25-00648-s001.pdf]

Supplementary Materials

of

Organocatalytic Asymmetric Aldol Reaction of Arylglyoxals and Hydroxyacetone: Enantioselective Synthesis of 2,3-Dihydroxy-1,4-Diones

Yu-Hao Zhou, Yu-Zu Zhang, Zhu-Lian Wu, Tian Cai, Wei Wen* and Qi-Xiang Guo*

Key Laboratory of Applied Chemistry of Chongqing Municipality, School of Chemistry and Chemical Engineering, Southwest University, Chongqing 400715, China
***** Correspondence: wenwei1989@swu.edu.cn; qxguo@swu.edu.cn.

1.Crystallographic data..2

2. Spectrums of ^{1}H NMR, ^{13}C NMR and HPLC..3

1. Crystallographic data

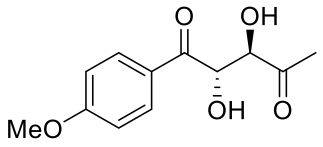

(2*S*,3*R*)-2,3-dihydroxy-1-(4-methoxyphenyl)pentane-1,4-dione

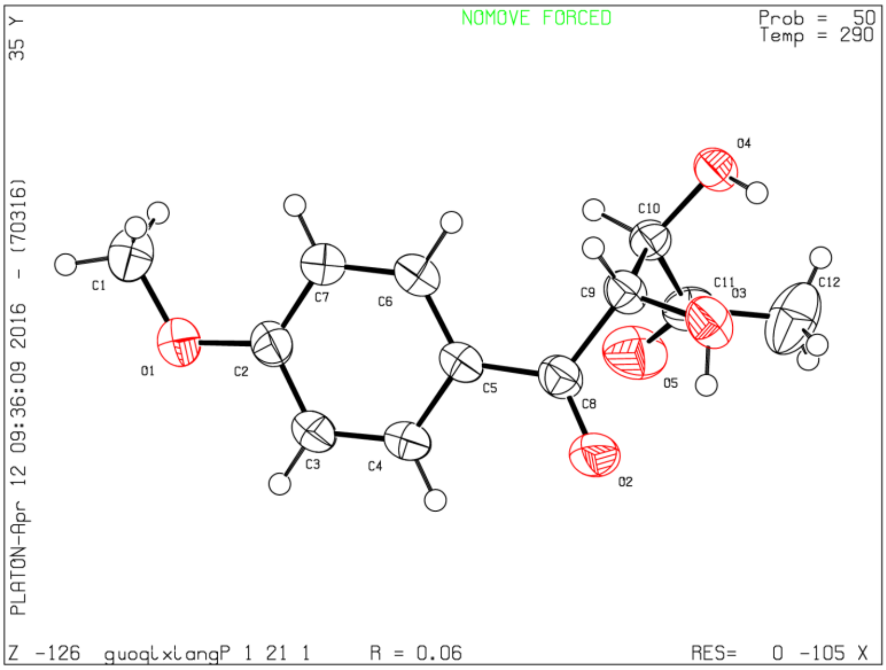

Chemical Formula	$C_{12}H_{14}O_5$
Formula weigh	238.23
Temperature	290.29(17)K
Wavelength	1.54184Å
Crystal system, space group	monoclinic, P 1 21 1
a/Å	6.4061(2)
b/Å	5.5861(2)
c/Å	16.4905(5)
α/°	90
β/°	91.091(3)
γ/ °	90
V/Å^3	590.01(4)
Z, Calculate density	2, 1.341g/cm^3

2. Spectrums of ^{1}H NMR, ^{13}C NMR and HPLC

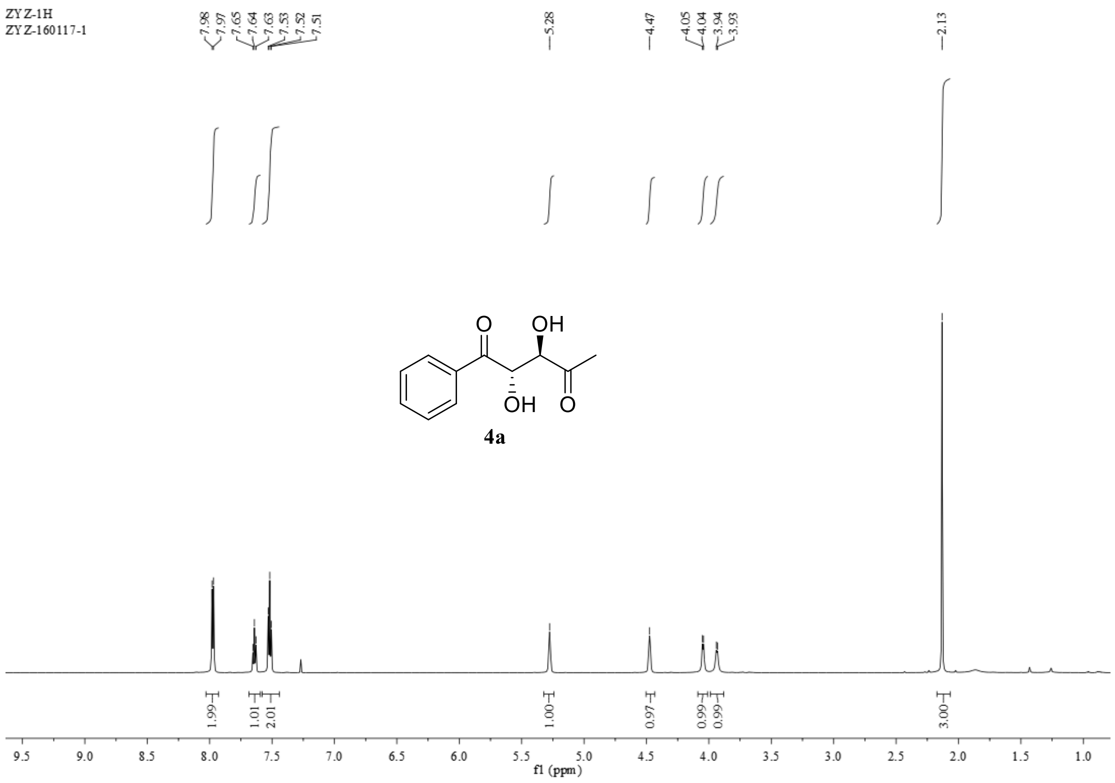

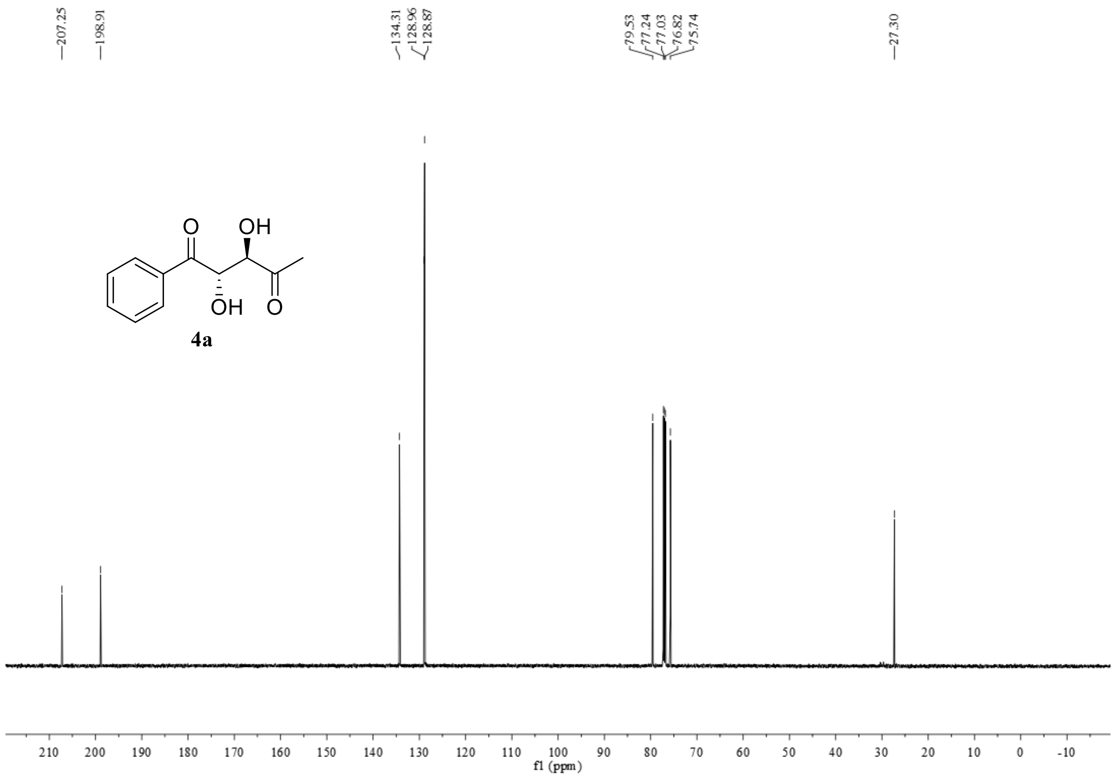

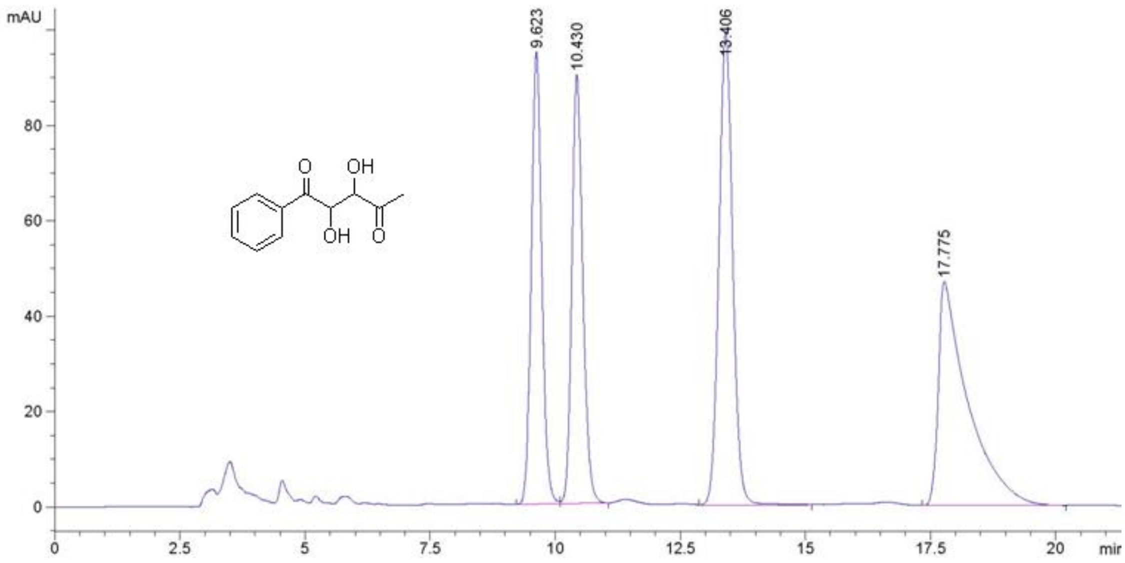

Peak #	RetTime [min]	Type	Width [min]	Area [mAU*s]	Height [mAU]	Area %
1	9.623	BV	0.2237	1379.68042	94.78895	21.0450
2	10.430	VB	0.2331	1368.21118	89.80470	20.8701
3	13.406	BB	0.2949	1898.57874	99.08701	28.9600
4	17.775	BB	0.5429	1909.38745	46.81028	29.1249

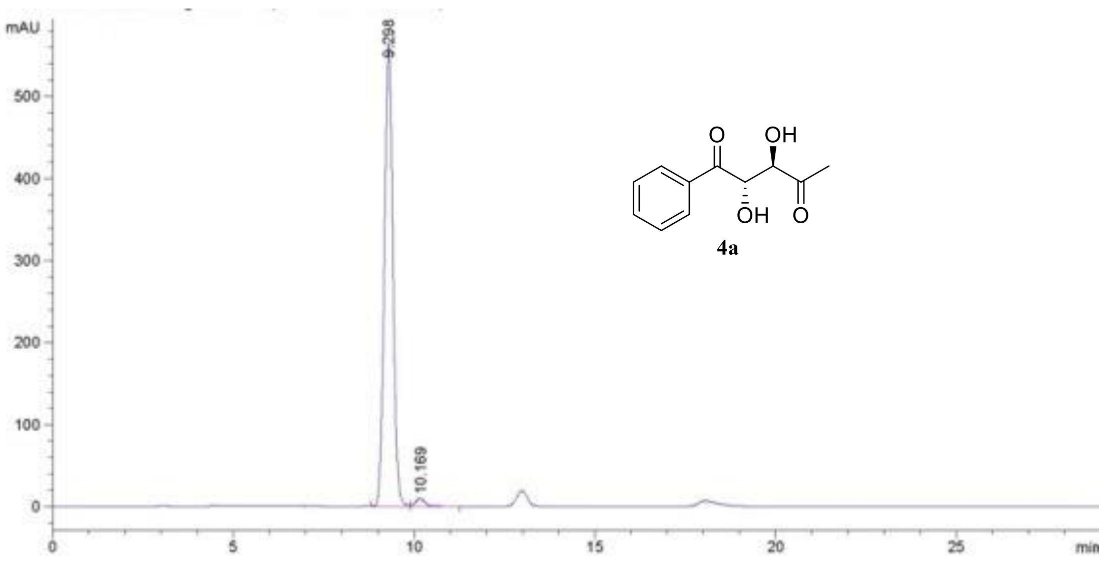

Peak #	RetTime [min]	Type	Width [min]	Area [mAU*s]	Height [mAU]	Area %
1	9.298	BV	0.2515	9231.80664	565.74780	98.1310
2	10.169	VB	0.2615	175.83051	10.09313	1.8690

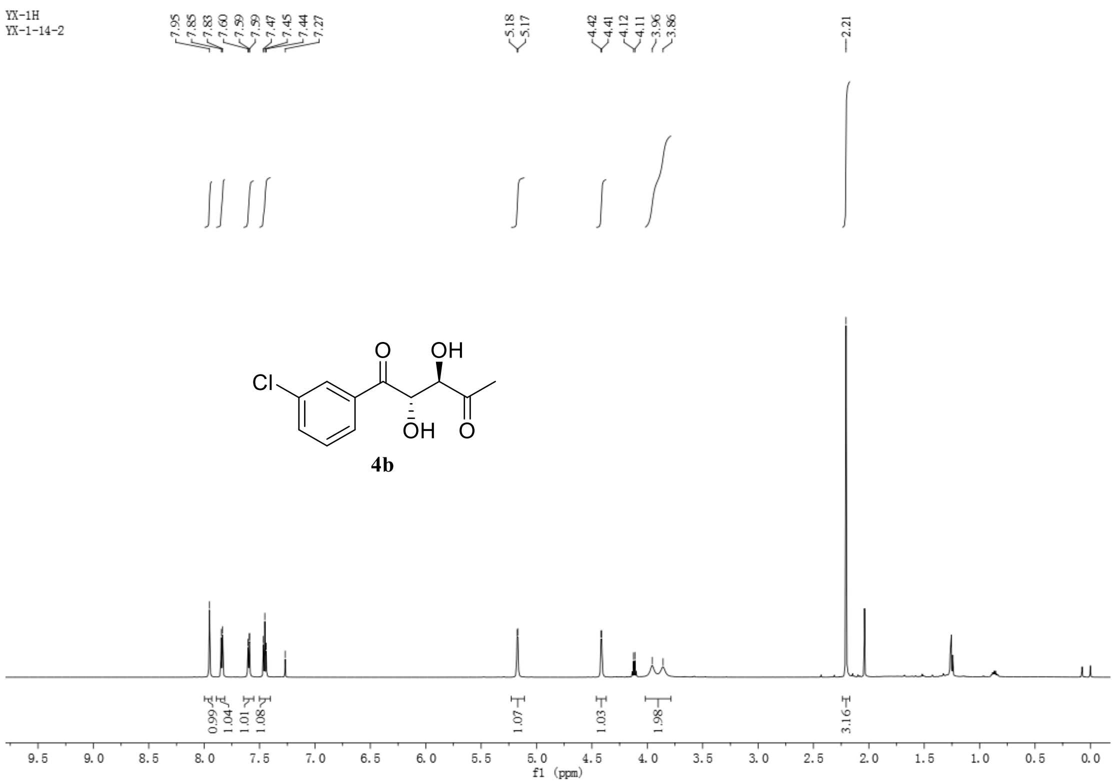
YX-1H
YX-1-14-2
7.95
7.85
7.83
7.60
7.59
7.59
7.47
7.45
7.44
7.27
5.18
5.17
4.42
4.41
4.12
4.11
3.96
3.86
2.21
Cl
O
OH
OH
O
4b
0.99
1.04
1.01
1.08
1.07
1.03
1.98
3.16
f1 (ppm)

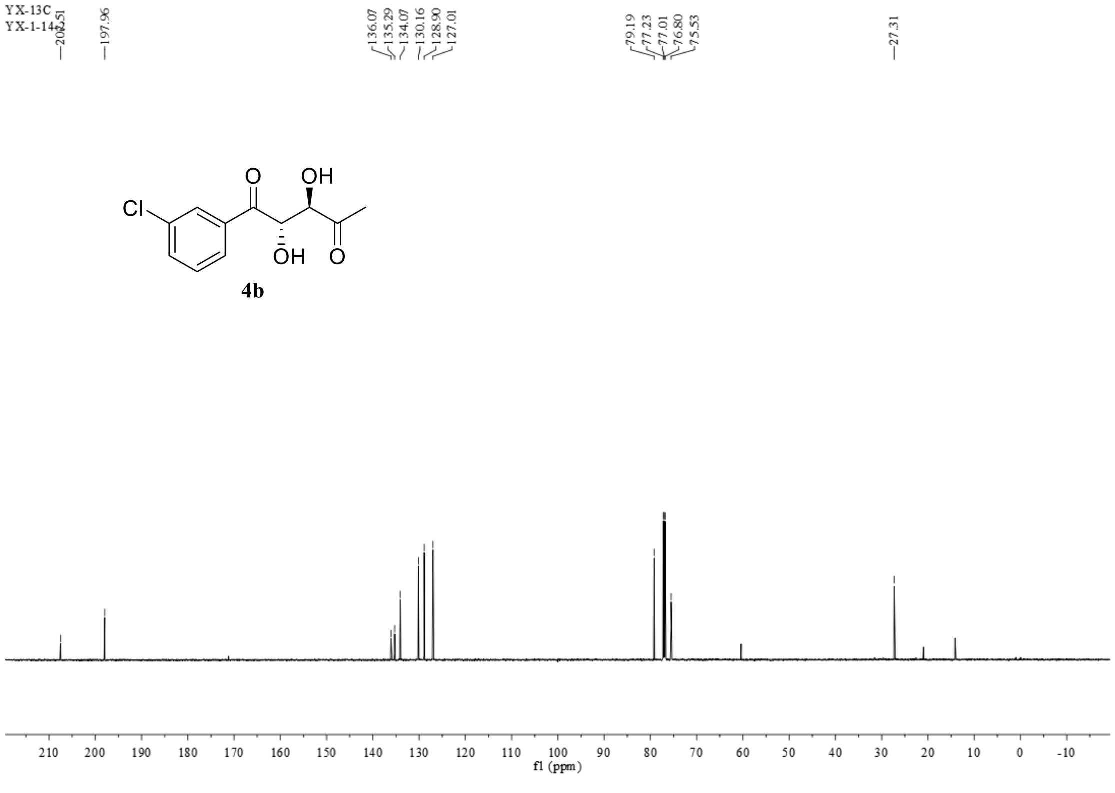
YX-13C
197.96
136.07
135.29
134.07
130.16
128.90
127.01
79.19
77.23
77.01
76.80
75.53
27.31
Cl
O
OH
OH
O
4b
f1 (ppm)

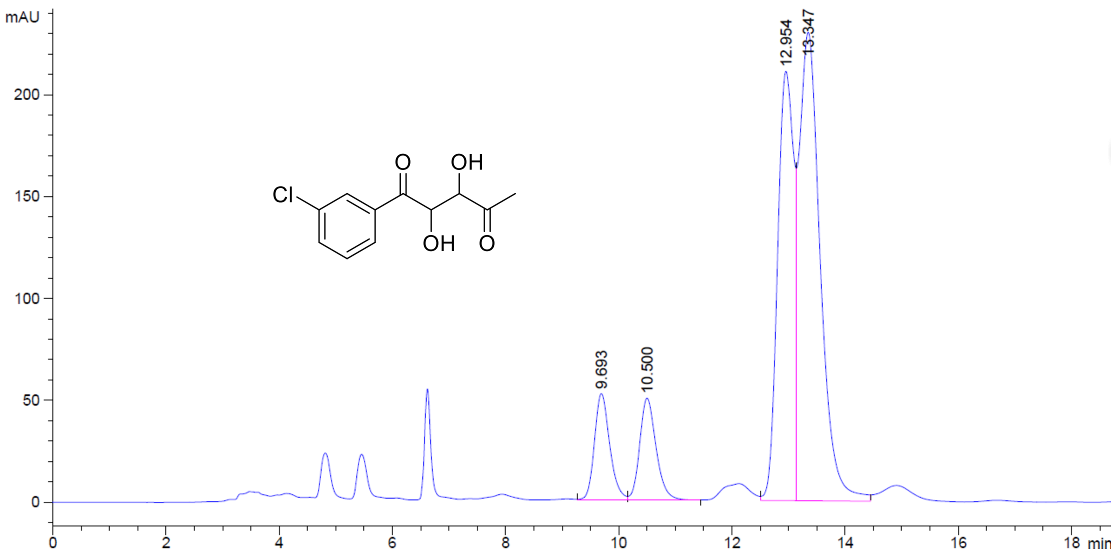

Peak #	RetTime [min]	Type	Width [min]	Area [mAU*s]	Height [mAU]	Area %
1	9.693	VV	0.2857	978.55841	52.21511	8.0325
2	10.500	VB	0.3076	1005.31732	49.97211	8.2521
3	12.954	VV	0.3038	4197.55957	210.78369	34.4556
4	13.347	VV	0.3832	6001.08203	229.97369	49.2598

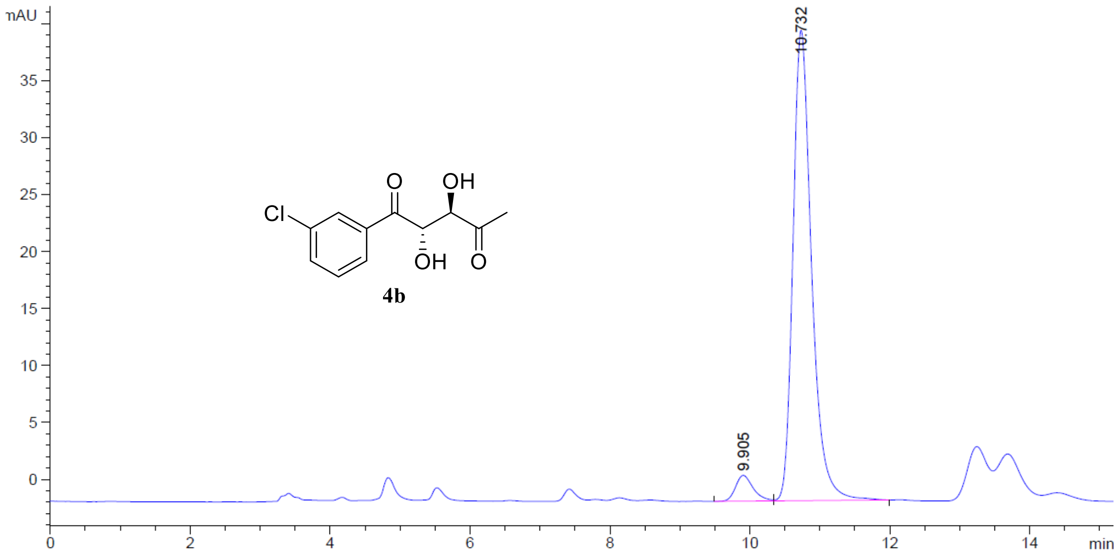

Peak #	RetTime [min]	Type	Width [min]	Area [mAU*s]	Height [mAU]	Area %
1	9.905	BV	0.2667	39.61375	2.26431	4.9358
2	10.732	VB	0.2810	762.96625	41.30092	95.0642

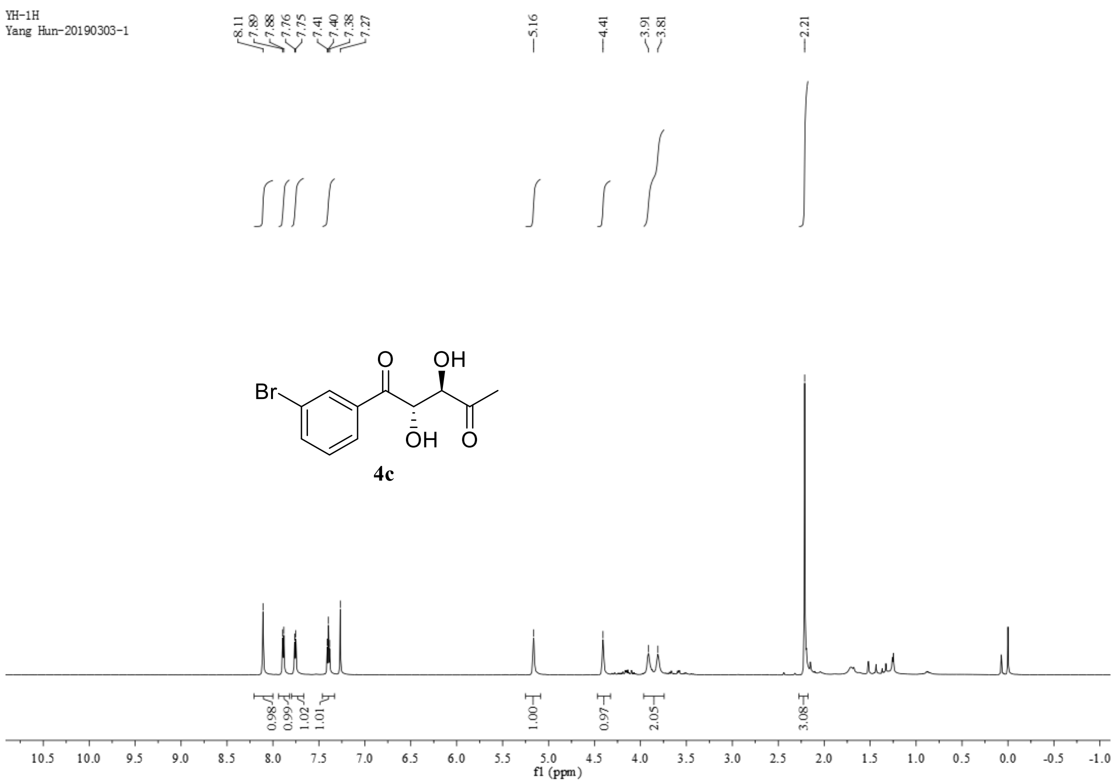
YH-1H
Yang Hun-20190303-1
8.11
7.89
7.88
7.76
7.75
7.41
7.40
7.38
7.27
5.16
4.41
3.91
3.81
2.21
Br
O
OH
OH
O
4c
0.98
0.99
1.02
1.01
1.00
0.97
2.05
3.08
10.5 10.0 9.5 9.0 8.5 8.0 7.5 7.0 6.5 6.0 5.5 5.0 4.5 4.0 3.5 3.0 2.5 2.0 1.5 1.0 0.5 0.0 -0.5 -1.0
f1 (ppm)

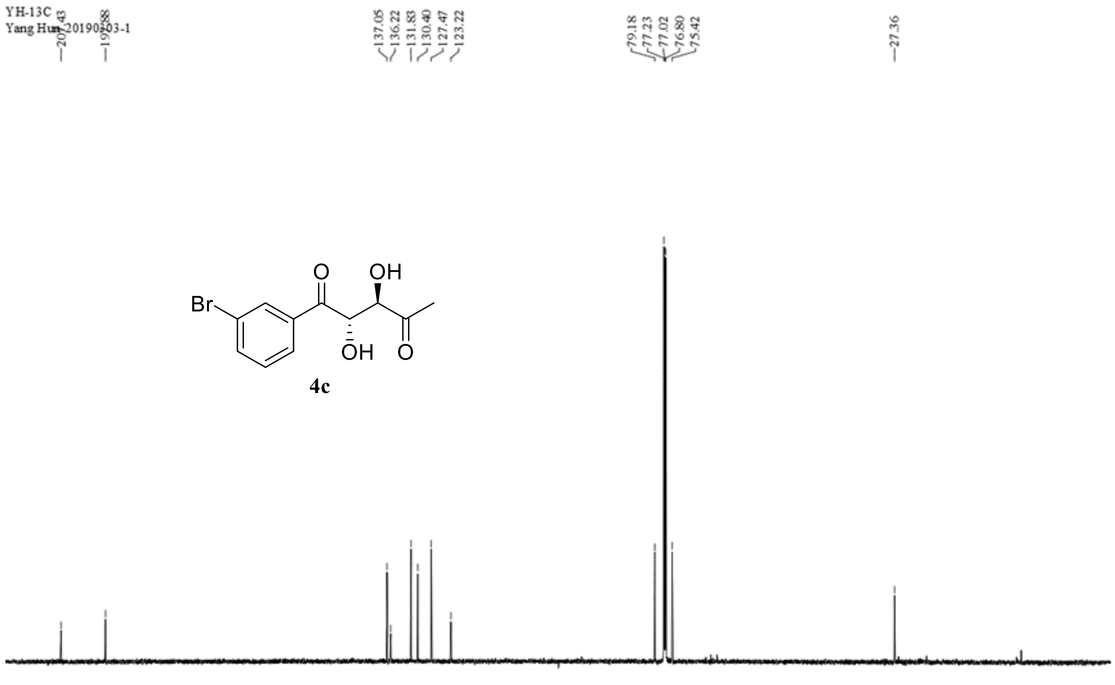
YH-13C
137.05
136.22
131.83
130.40
127.47
123.22
79.18
77.23
77.02
76.80
75.42
27.36
Br
O
OH
OH
O
4c
210 200 190 180 170 160 150 140 130 120 110 100 90 80 70 60 50 40 30 20 10 0 -10
f1 (ppm)

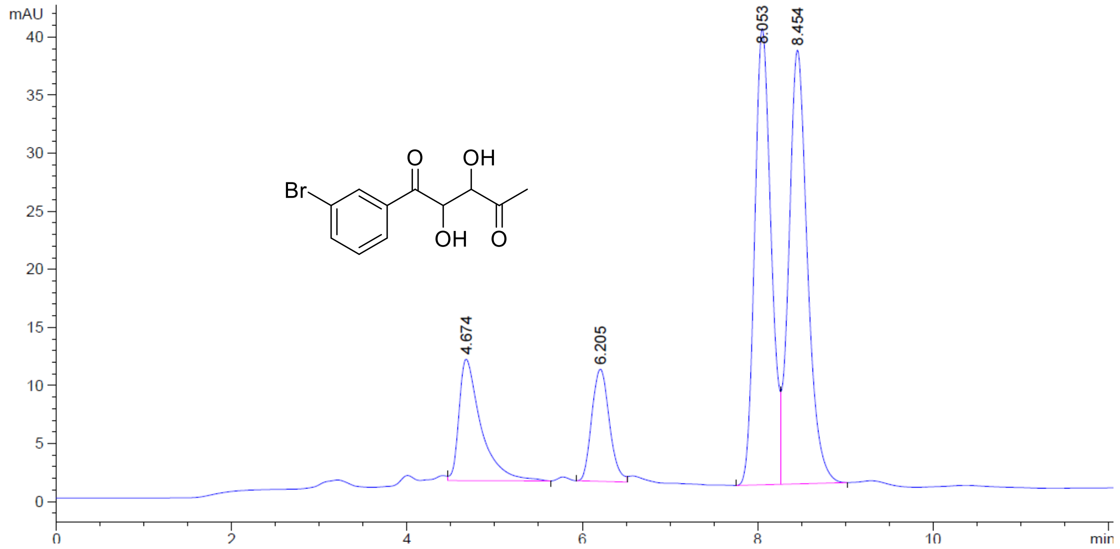

Peak #	RetTime [min]	Type	Width [min]	Area [mAU*s]	Height [mAU]	Area %
1	4.674	VB	0.2561	182.46974	10.45192	13.2110
2	6.205	BV	0.2211	134.90280	9.66418	9.7671
3	8.053	BV	0.1999	511.22772	39.28375	37.0133
4	8.454	VB	0.2236	552.60016	37.35242	40.0087

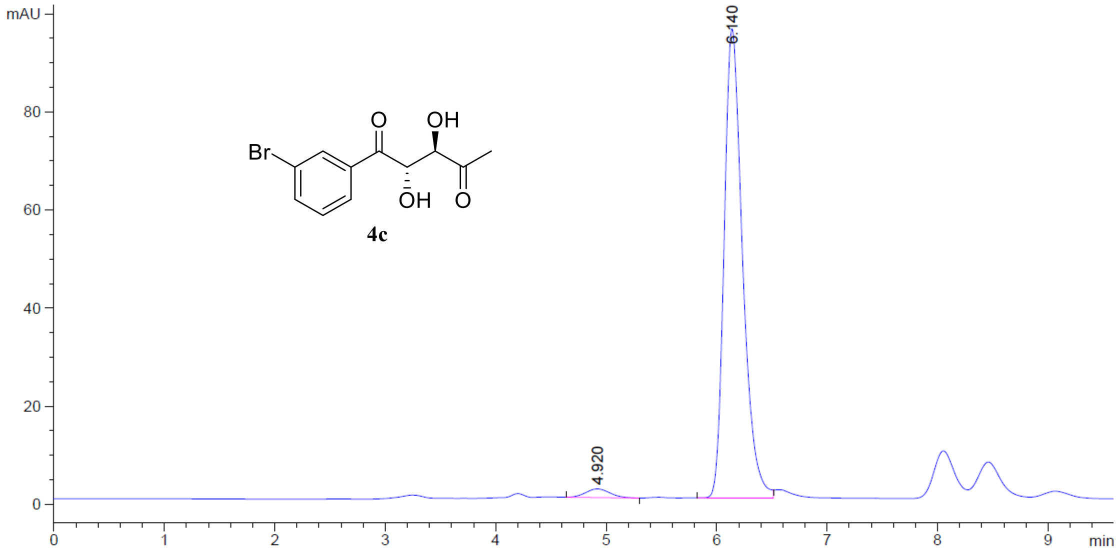

Peak #	RetTime [min]	Type	Width [min]	Area [mAU*s]	Height [mAU]	Area %
1	4.920	BB	0.2383	27.06308	1.76732	2.3814
2	6.140	BV	0.1761	1109.36414	95.64050	97.6186

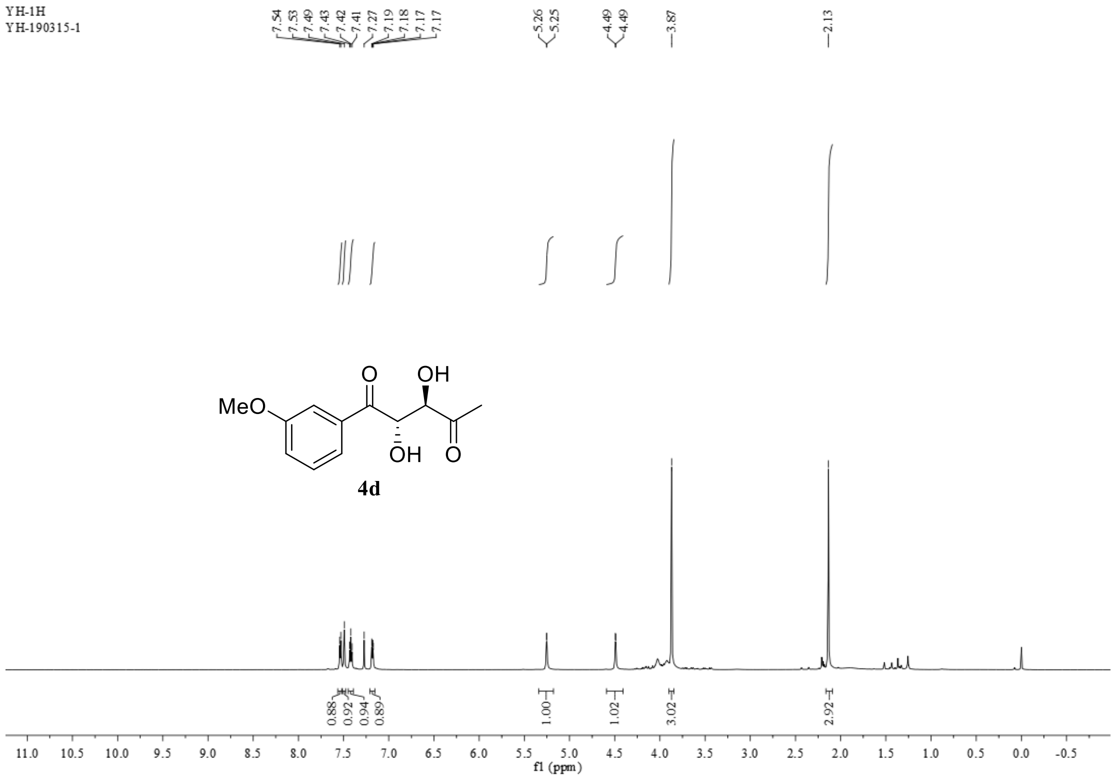
YH-1H
YH-190315-1
7.54
7.53
7.49
7.43
7.42
7.41
7.27
7.19
7.18
7.17
7.17
5.26
5.25
4.49
4.49
3.87
2.13
MeO
O
OH
OH
O
4d
0.88
0.92
0.94
0.89
1.00
1.02
3.02
2.92
11.0 10.5 10.0 9.5 9.0 8.5 8.0 7.5 7.0 6.5 6.0 5.5 5.0 4.5 4.0 3.5 3.0 2.5 2.0 1.5 1.0 0.5 0.0 -0.5
f1 (ppm)

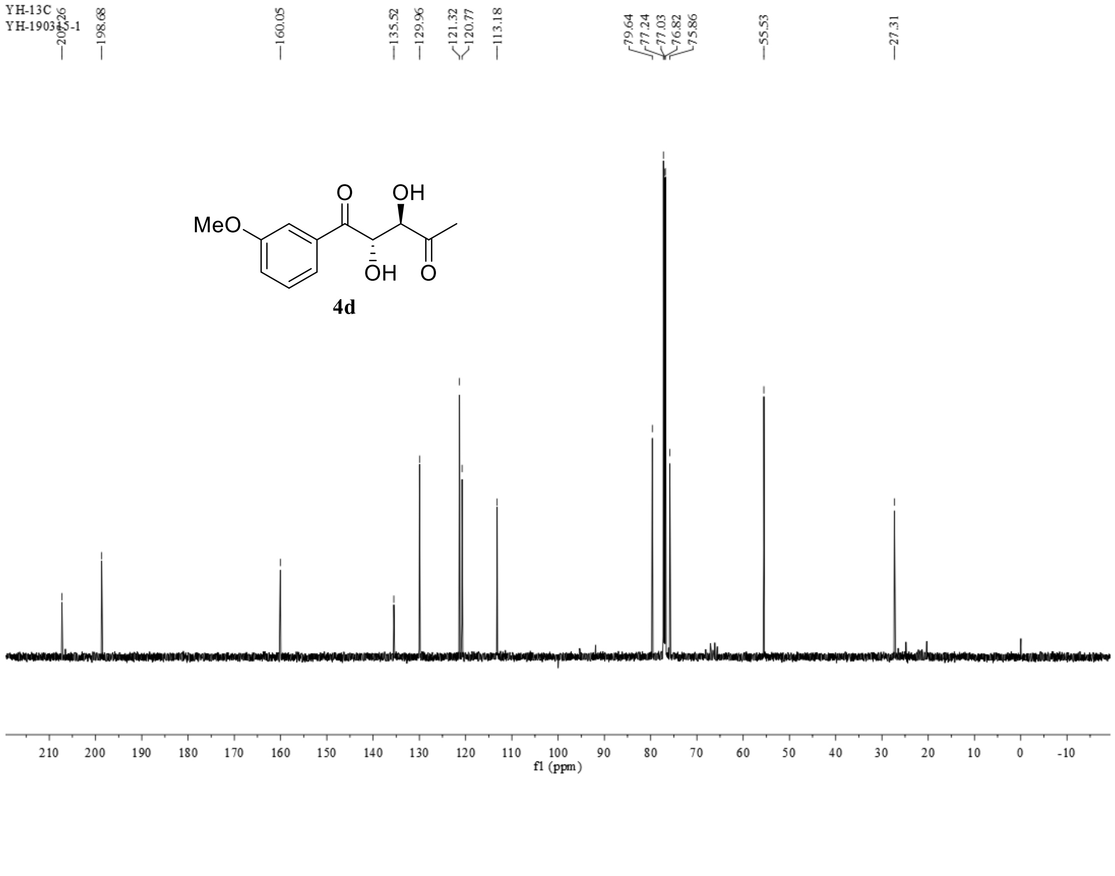
YH-13C
YH-190315-1
198.68
160.05
135.52
129.96
121.32
120.77
113.18
79.64
77.24
77.03
76.82
75.86
55.53
27.31
MeO
O
OH
OH
O
4d
210 200 190 180 170 160 150 140 130 120 110 100 90 80 70 60 50 40 30 20 10 0 -10
f1 (ppm)

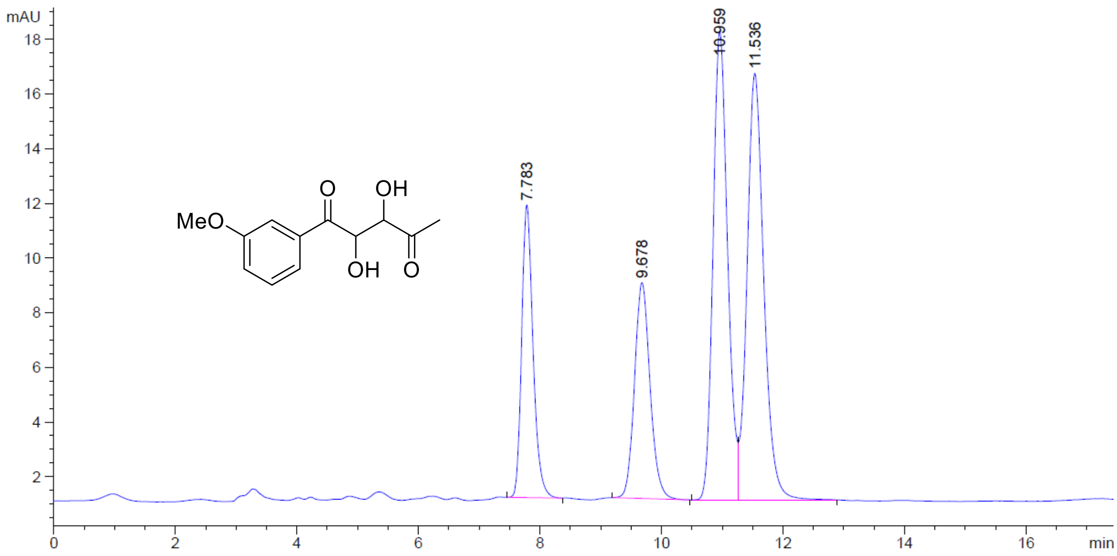

Peak #	RetTime [min]	Type	Width [min]	Area [mAU*s]	Height [mAU]	Area %
1	7.783	BB	0.1979	138.89966	10.70775	16.0708
2	9.678	BB	0.2731	140.54344	7.89927	16.2609
3	10.959	BV	0.2551	284.94641	17.14184	32.9684
4	11.536	VB	0.2926	299.91089	15.60809	34.6998

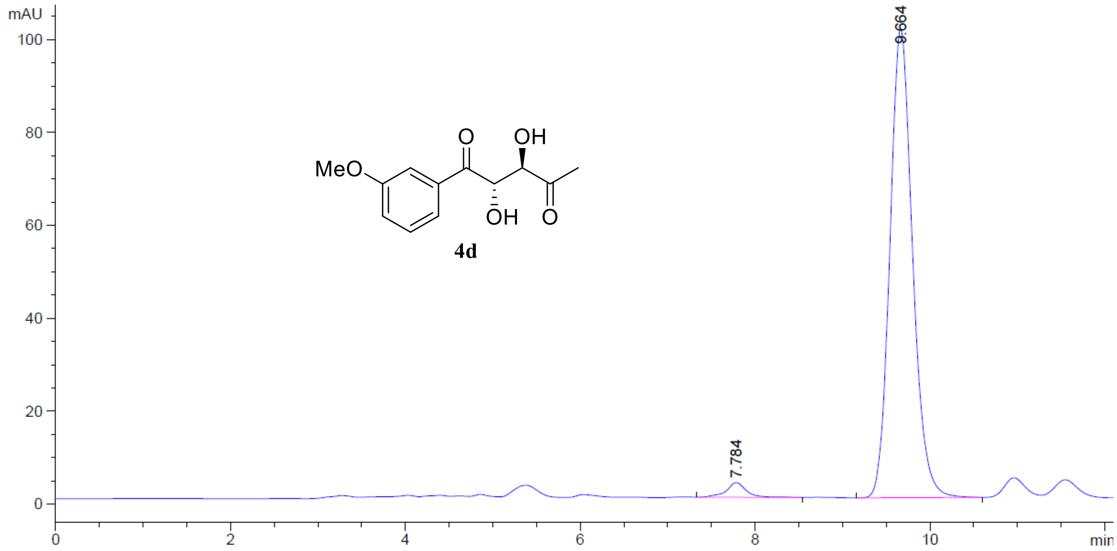

Peak #	RetTime [min]	Type	Width [min]	Area [mAU*s]	Height [mAU]	Area %
1	7.784	BB	0.2325	50.58262	3.15048	2.7155
2	9.664	BB	0.2749	1812.14502	100.96157	97.2845

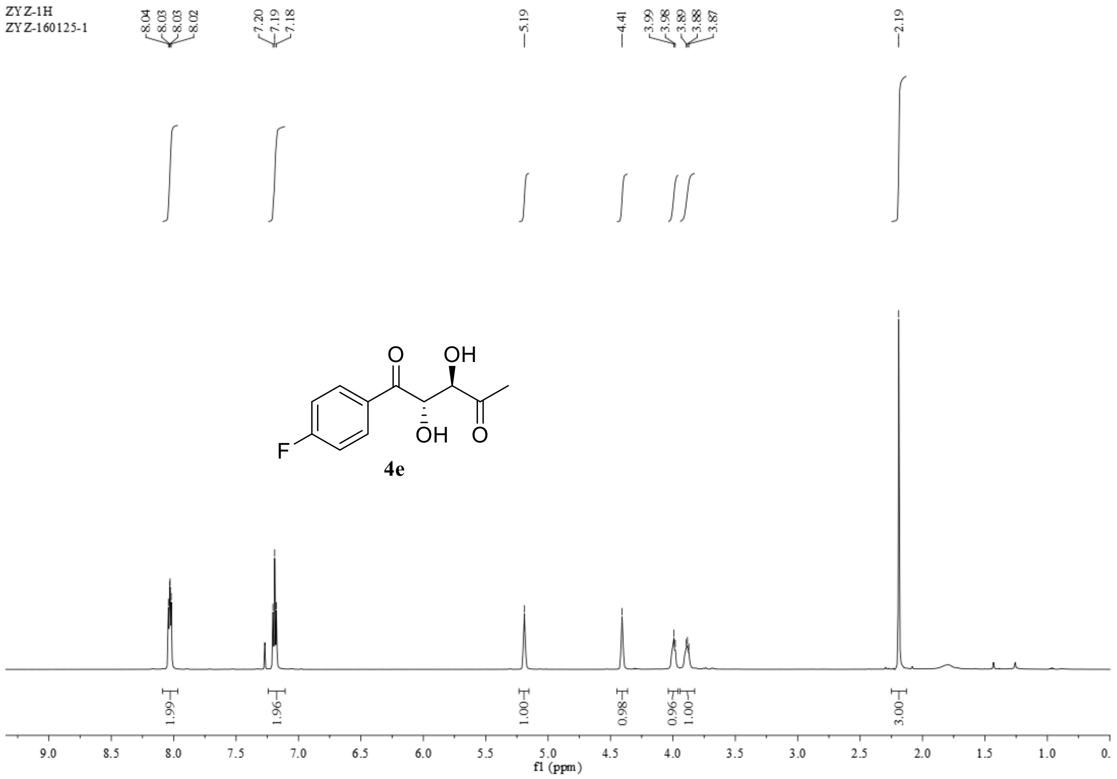
ZYZ-1H
ZYZ-160125-1
8.04
8.03
8.03
8.02
7.20
7.19
7.18
5.19
4.41
3.99
3.98
3.89
3.88
3.87
2.19
4e
1.99
1.96
1.00
0.98
0.96
1.00
3.00
f1 (ppm)

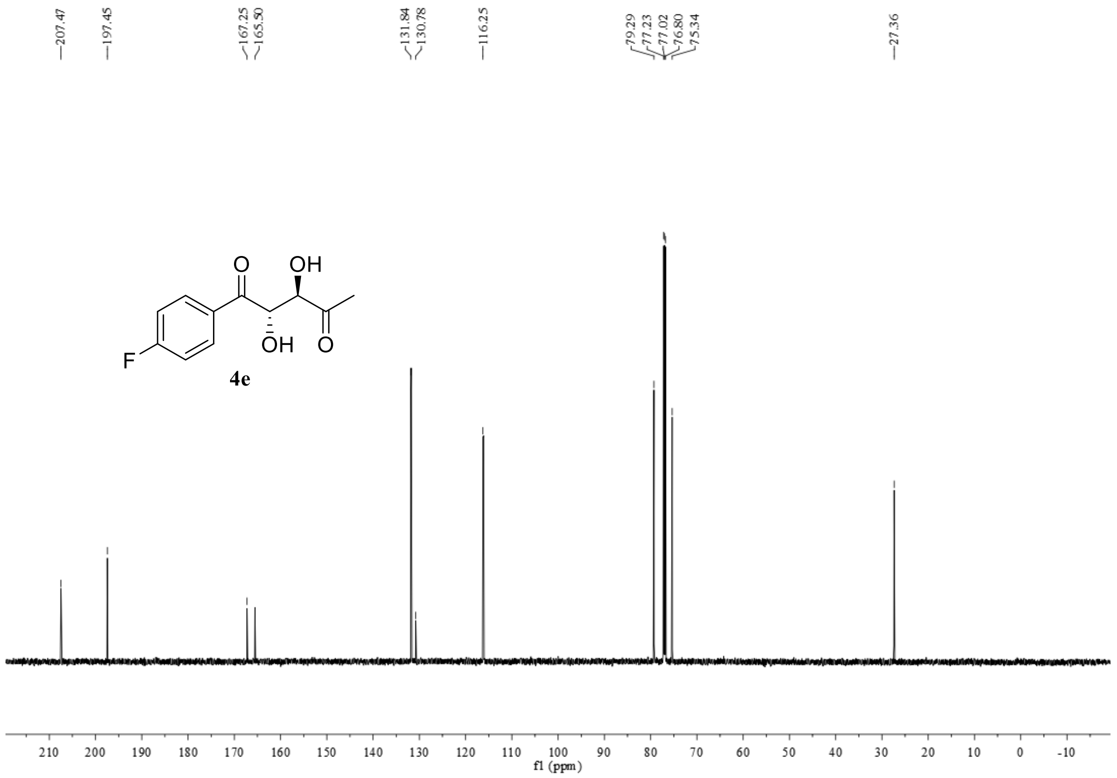
207.47
197.45
167.25
165.50
131.84
130.78
116.25
79.29
77.23
77.02
76.80
75.34
27.36
4e
f1 (ppm)

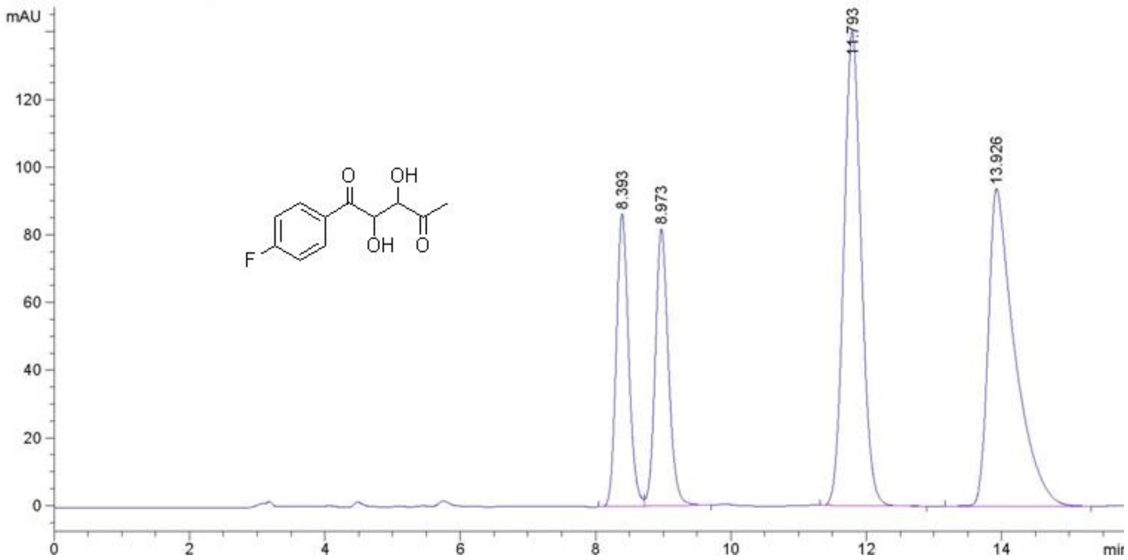

Peak #	RetTime [min]	Type	Width [min]	Area [mAU*s]	Height [mAU]	Area %
1	8.393	BV	0.1956	1092.69885	86.41489	15.0804
2	8.973	VB	0.2066	1100.52393	81.74480	15.1884
3	11.793	BB	0.2744	2508.90356	140.10246	34.6254
4	13.926	BB	0.3834	2543.71313	93.77689	35.1058

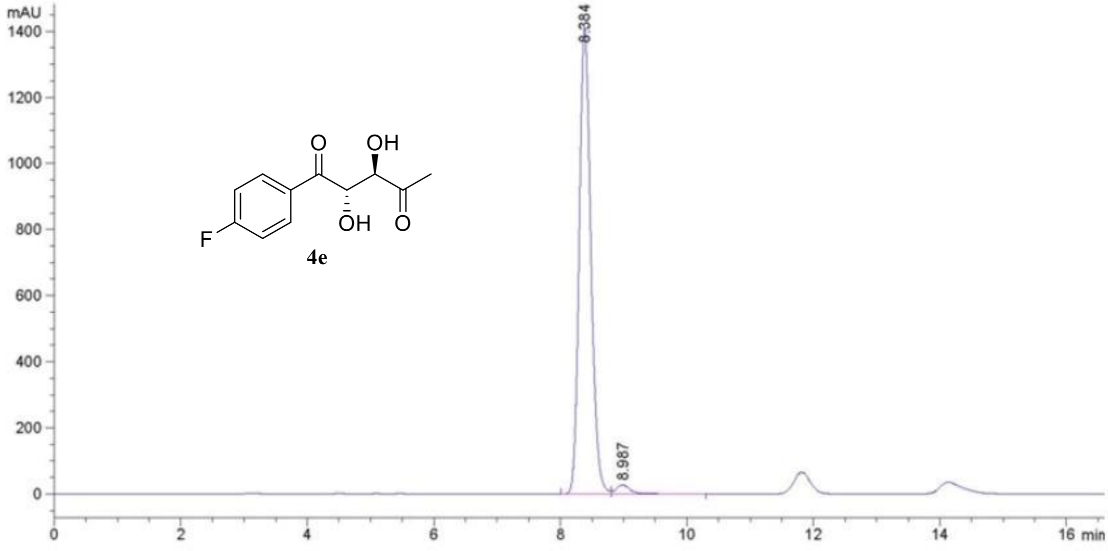

Peak #	RetTime [min]	Type	Width [min]	Area [mAU*s]	Height [mAU]	Area %
1	8.384	BV	0.1965	1.79747e4	1412.65088	97.7381
2	8.987	VB	0.2273	415.98679	27.07613	2.2619

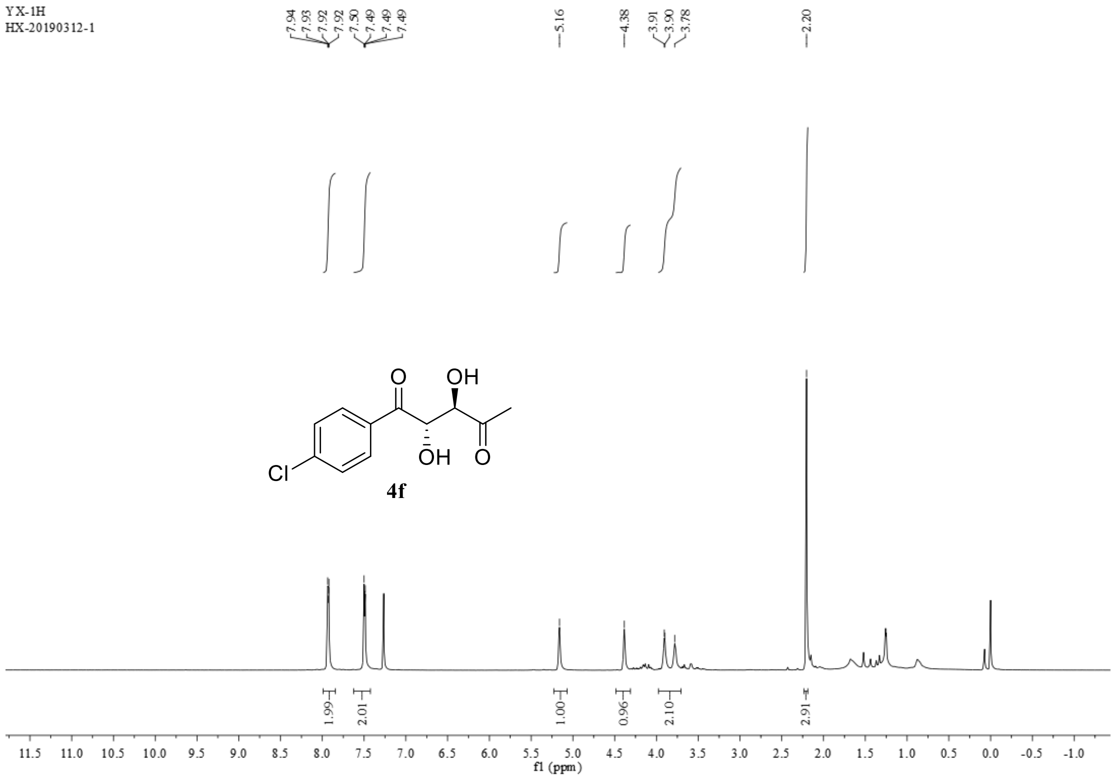
YX-1H
HX-20190312-1
7.94
7.93
7.92
7.92
7.50
7.49
7.49
7.49
5.16
4.38
3.91
3.90
3.78
2.20
O
OH
Cl
OH
O
4f
1.99
2.01
1.00
0.96
2.10
2.91
11.5 11.0 10.5 10.0 9.5 9.0 8.5 8.0 7.5 7.0 6.5 6.0 5.5 5.0 4.5 4.0 3.5 3.0 2.5 2.0 1.5 1.0 0.5 0.0 -0.5 -1.0
f1 (ppm)

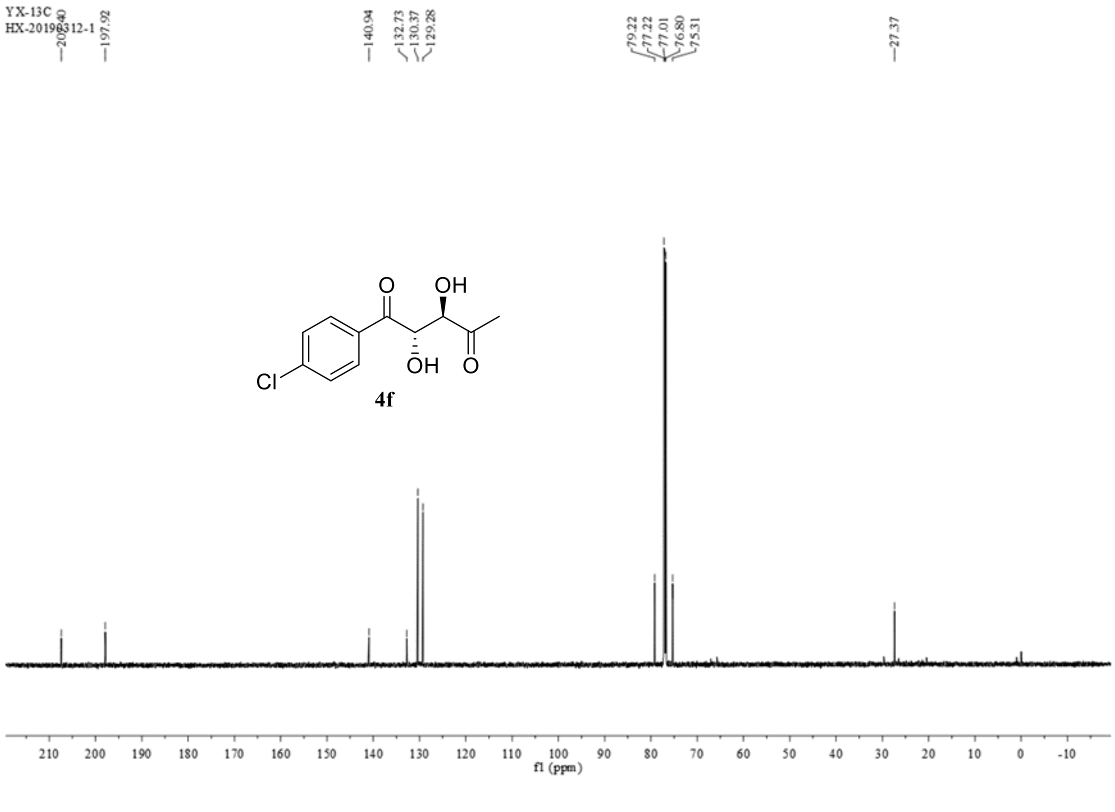
YX-13C
HX-20190312-1
197.92
140.94
132.73
130.37
129.28
79.22
77.22
77.01
76.80
75.31
27.37
O
OH
Cl
OH
O
4f
210 200 190 180 170 160 150 140 130 120 110 100 90 80 70 60 50 40 30 20 10 0 -10
f1 (ppm)

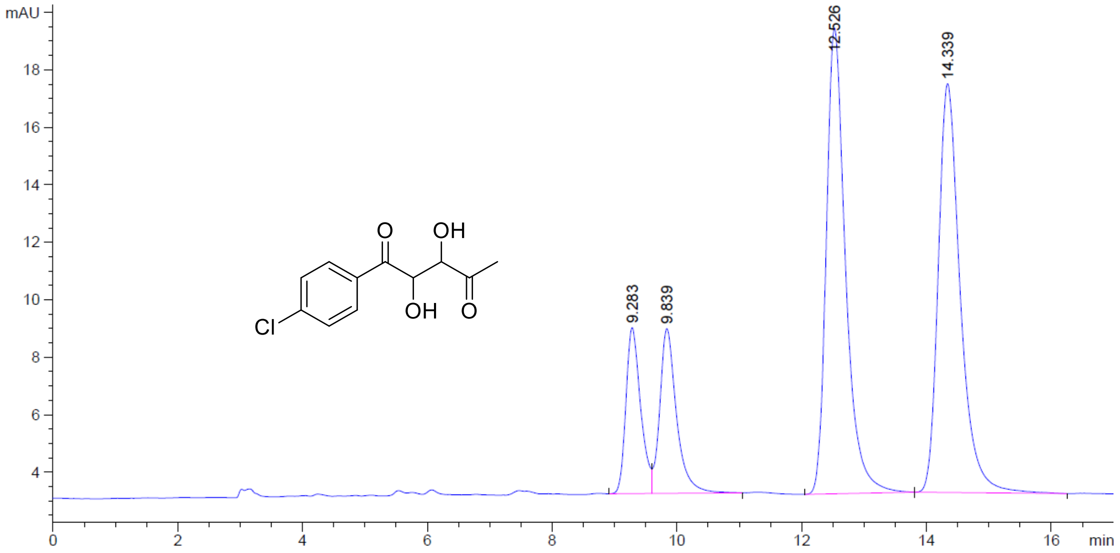

Peak #	RetTime [min]	Type	Width [min]	Area [mAU*s]	Height [mAU]	Area %
1	9.283	BV	0.2480	94.63755	5.77450	10.5309
2	9.839	VB	0.2757	106.86500	5.73339	11.8915
3	12.526	BB	0.3217	347.79559	16.20674	38.7014
4	14.339	BB	0.3695	349.36639	14.22785	38.8762

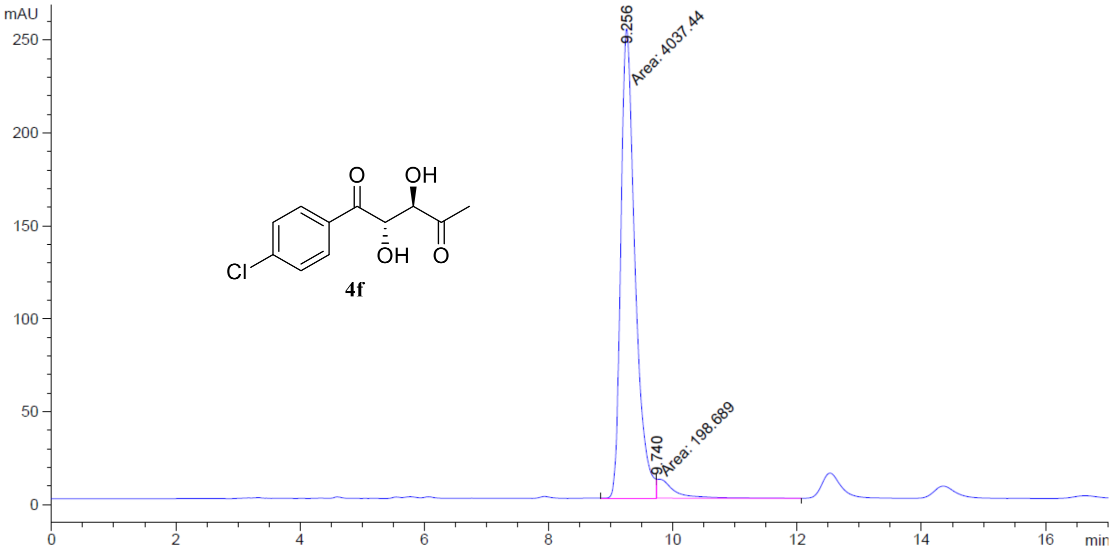

Peak #	RetTime [min]	Type	Width [min]	Area [mAU*s]	Height [mAU]	Area %
1	9.256	MF	0.2664	4037.43823	252.56102	95.3097
2	9.740	FM	0.3238	198.68918	10.22630	4.6903

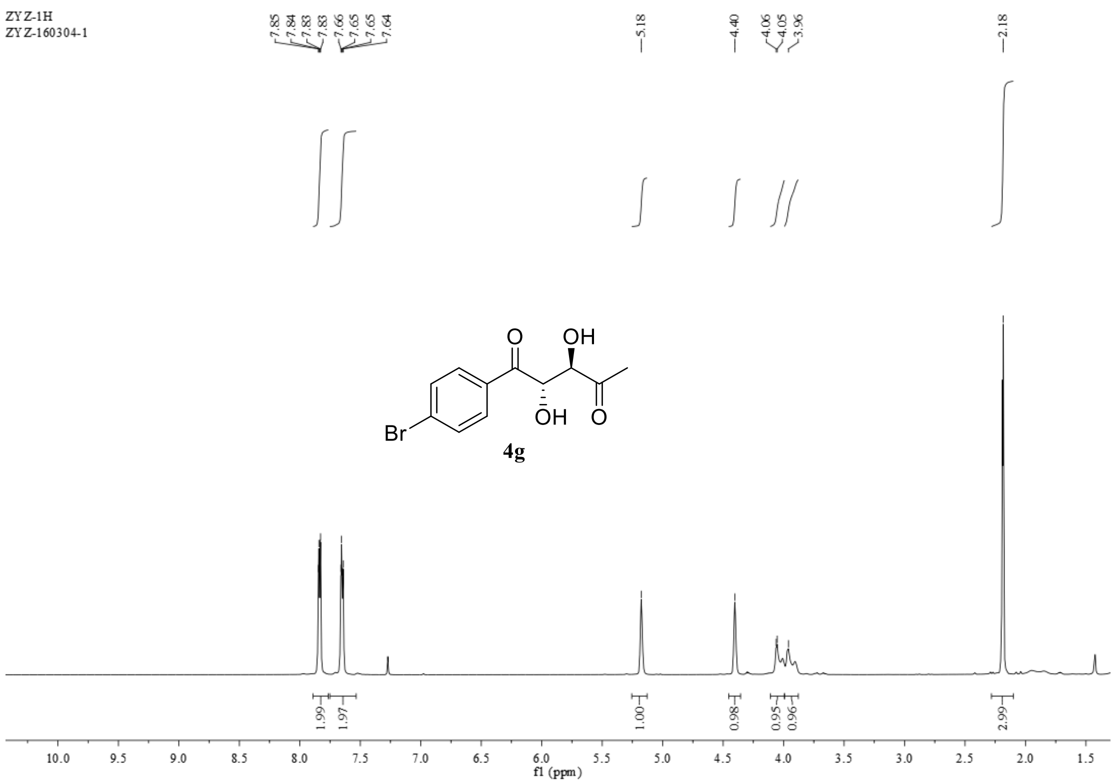
ZYZ-1H
ZYZ-160304-1
7.85
7.84
7.83
7.83
7.66
7.65
7.65
7.64
5.18
4.40
4.06
4.05
3.96
2.18
Br
O
OH
OH
O
4g
1.99
1.97
1.00
0.98
0.95
0.96
2.99
f1 (ppm)

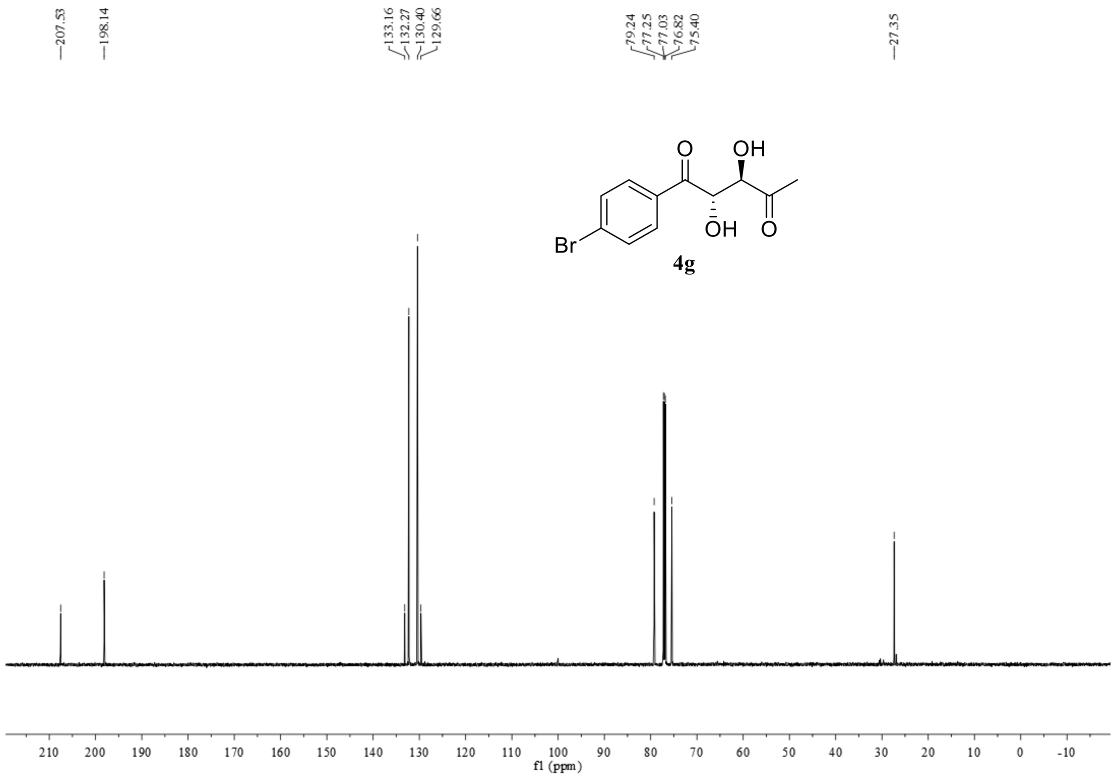
207.53
198.14
133.16
132.27
130.40
129.66
79.24
77.25
77.03
76.82
75.40
27.35
Br
O
OH
OH
O
4g
f1 (ppm)

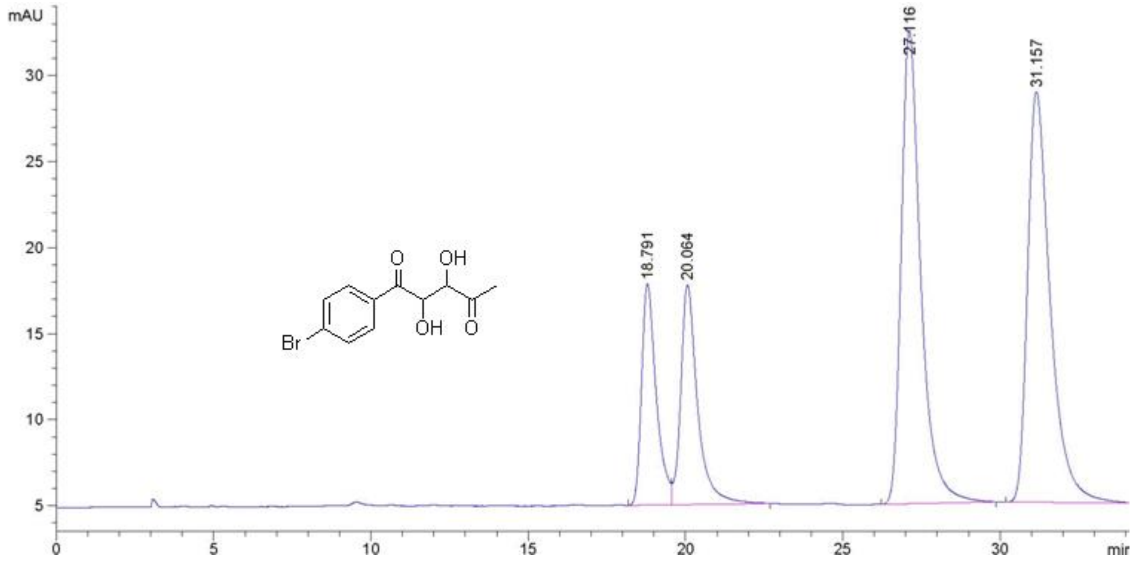

Peak #	RetTime [min]	Type	Width [min]	Area [mAU*s]	Height [mAU]	Area %
1	18.791	BV	0.4838	416.38272	12.86677	12.7201
2	20.064	VB	0.5456	473.31842	12.78037	14.4594
3	27.116	BB	0.6450	1188.08020	27.51219	36.2947
4	31.157	BBA	0.7590	1195.64465	23.87403	36.5258

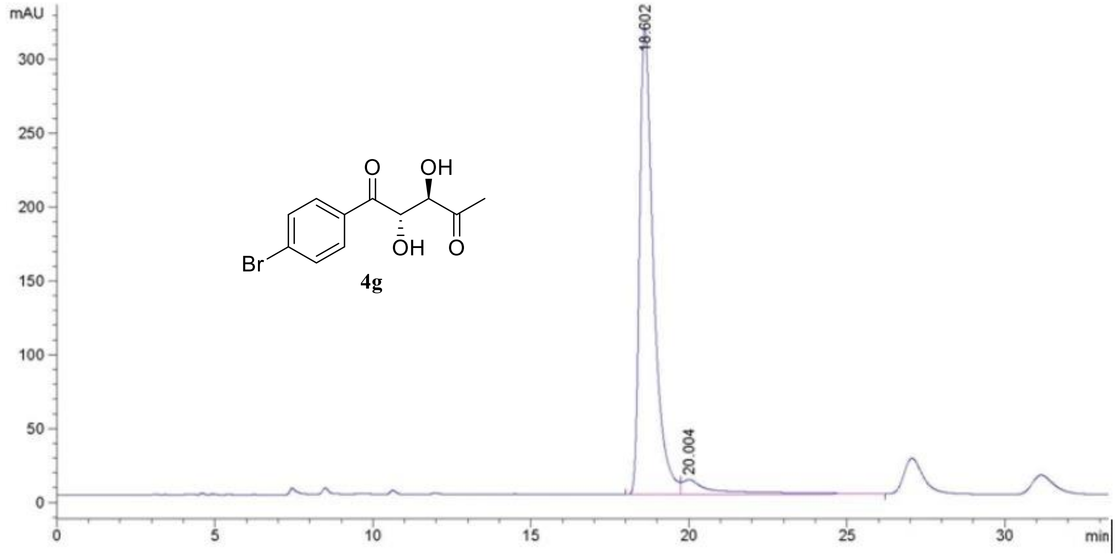

Peak #	RetTime [min]	Type	Width [min]	Area [mAU*s]	Height [mAU]	Area %
1	18.602	BV	0.4558	9624.17090	315.97885	93.1760
2	20.004	VB	0.9249	704.85004	9.97487	6.8240

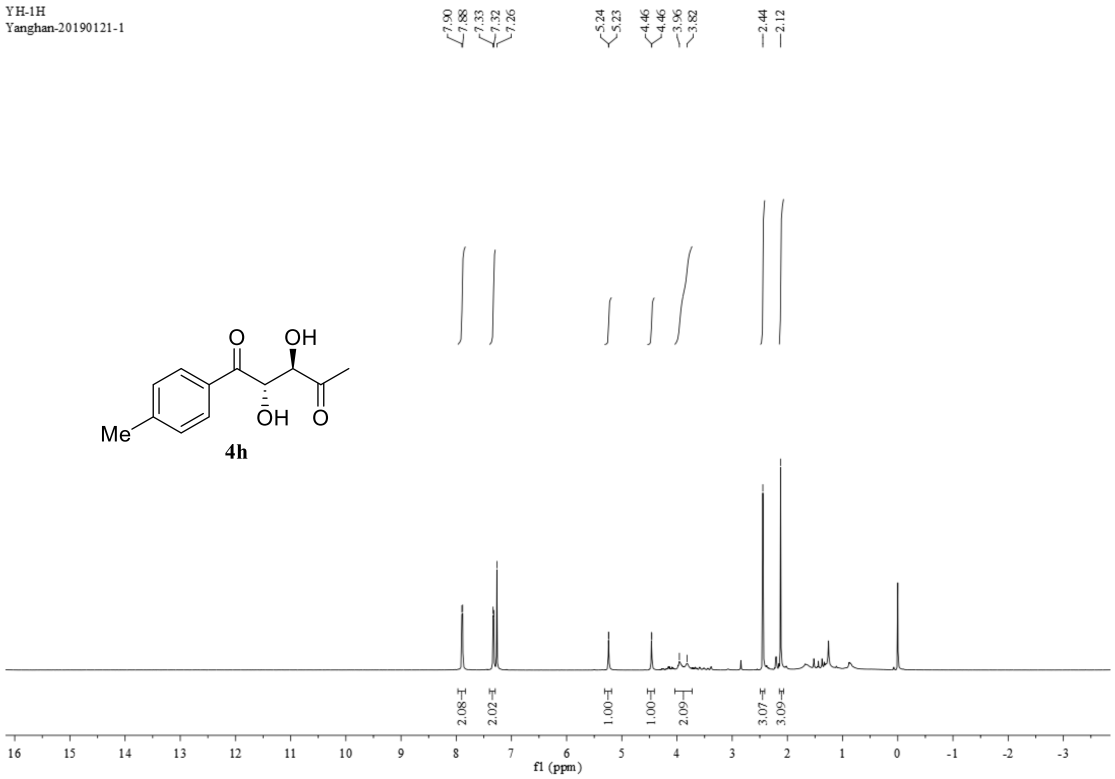
YH-1H
Yanghan-20190121-1
7.90
7.88
7.33
7.32
7.26
5.24
5.23
4.46
4.46
3.96
3.82
2.44
2.12
O
OH
OH
O
Me
4h
2.08
2.02
1.00
1.00
2.09
3.07
3.09
16 15 14 13 12 11 10 9 8 7 6 5 4 3 2 1 0 -1 -2 -3
f1 (ppm)

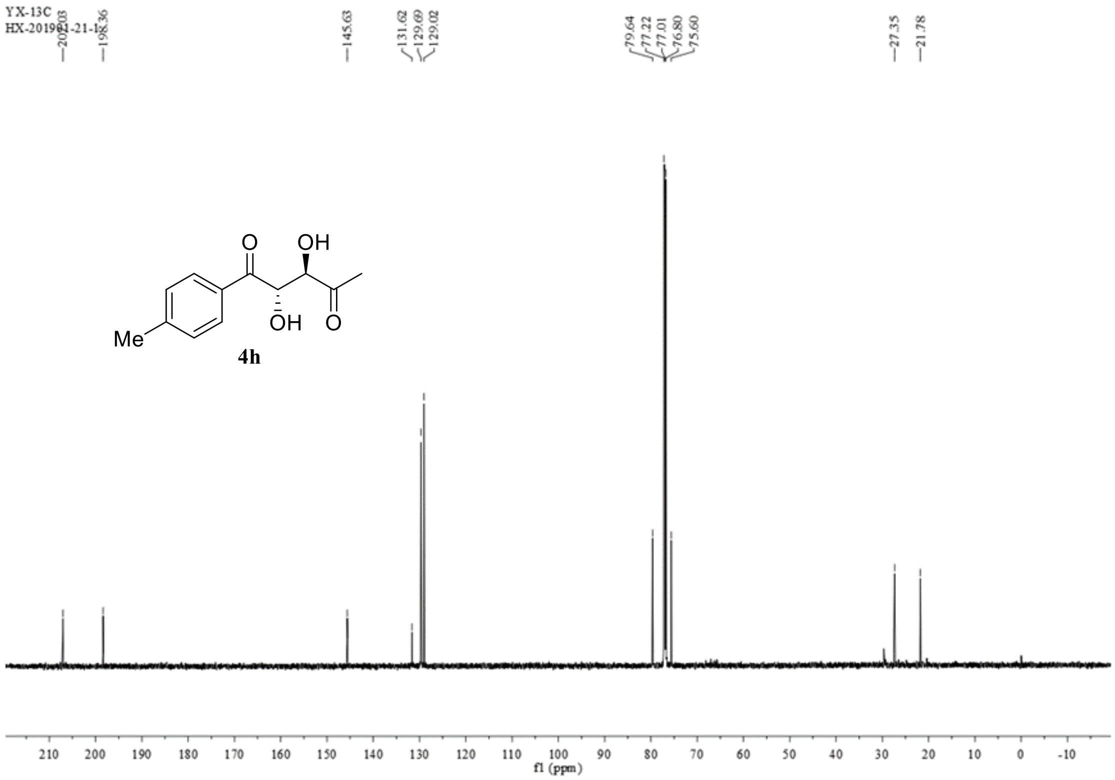
YX-13C
198.36
145.63
131.62
129.69
129.02
79.64
77.22
77.01
76.80
75.60
27.35
21.78
O
OH
OH
O
Me
4h
210 200 190 180 170 160 150 140 130 120 110 100 90 80 70 60 50 40 30 20 10 0 -10
f1 (ppm)

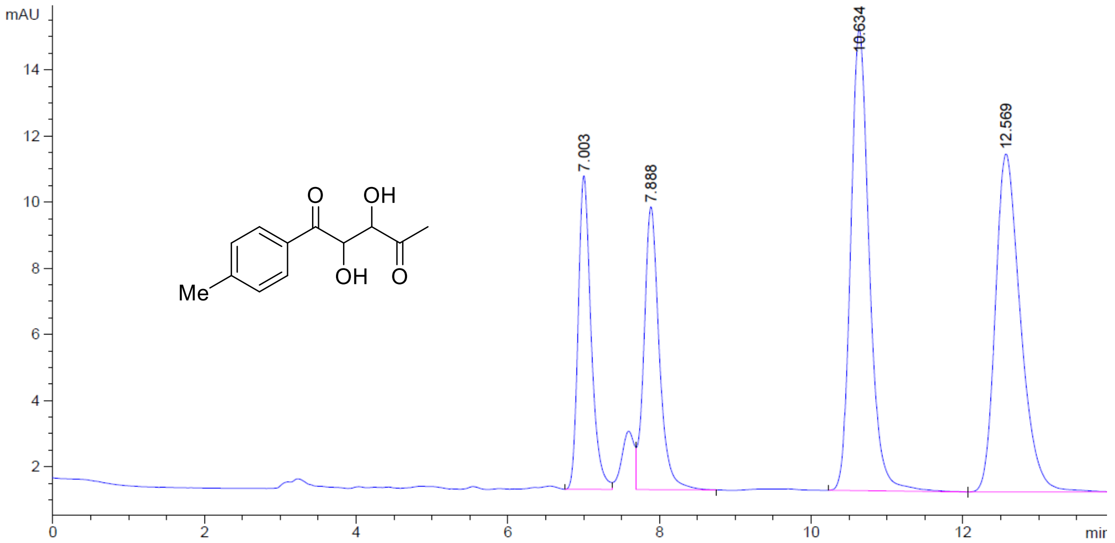

Peak #	RetTime [min]	Type	Width [min]	Area [mAU*s]	Height [mAU]	Area %
1	7.003	BV	0.1785	111.91376	9.48060	15.9151
2	7.888	VB	0.2077	119.19920	8.55379	16.9512
3	10.634	BB	0.2595	237.23840	13.95552	33.7374
4	12.569	BBA	0.3496	234.83995	10.22251	33.3963

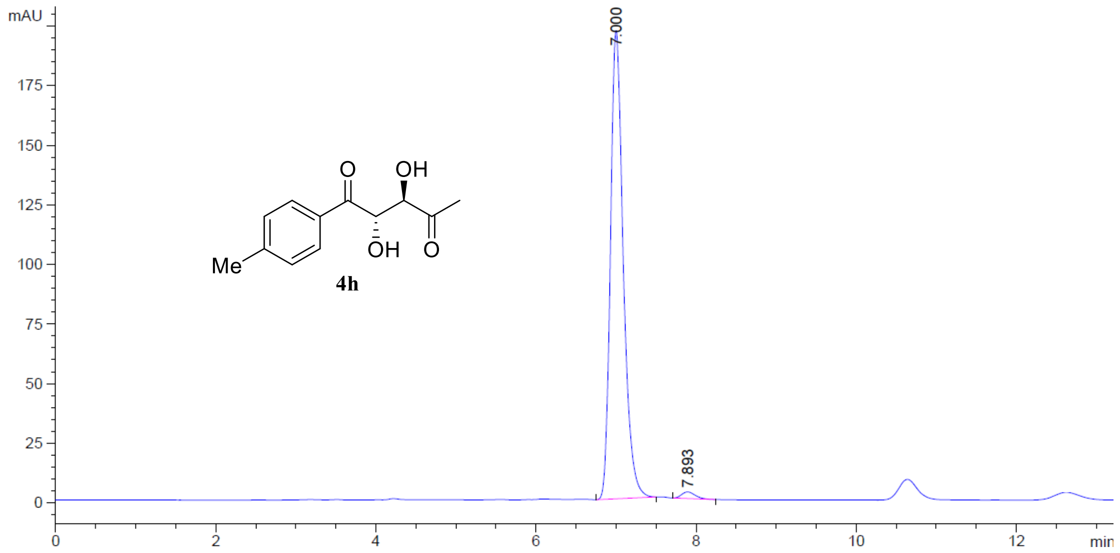

Peak #	RetTime [min]	Type	Width [min]	Area [mAU*s]	Height [mAU]	Area %
1	7.000	BB	0.1689	2181.36084	196.41527	98.5299
2	7.893	BB	0.1812	32.54693	2.79137	1.4701

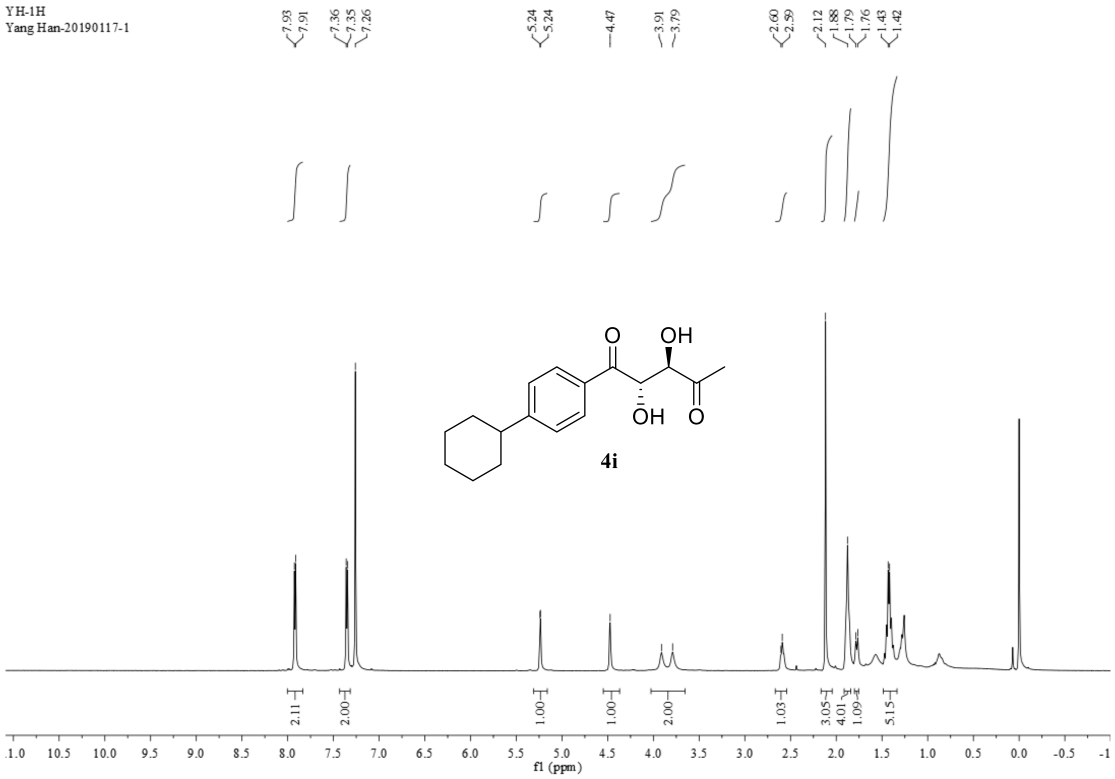
YH-1H
Yang Han-20190117-1
7.93
7.91
7.36
7.35
7.26
5.24
5.24
4.47
3.91
3.79
2.60
2.59
2.12
1.88
1.79
1.76
1.43
1.42
O
OH
OH
O
4i
2.11
2.00
1.00
1.00
2.00
1.03
3.05
4.01
1.09
5.15
f1 (ppm)

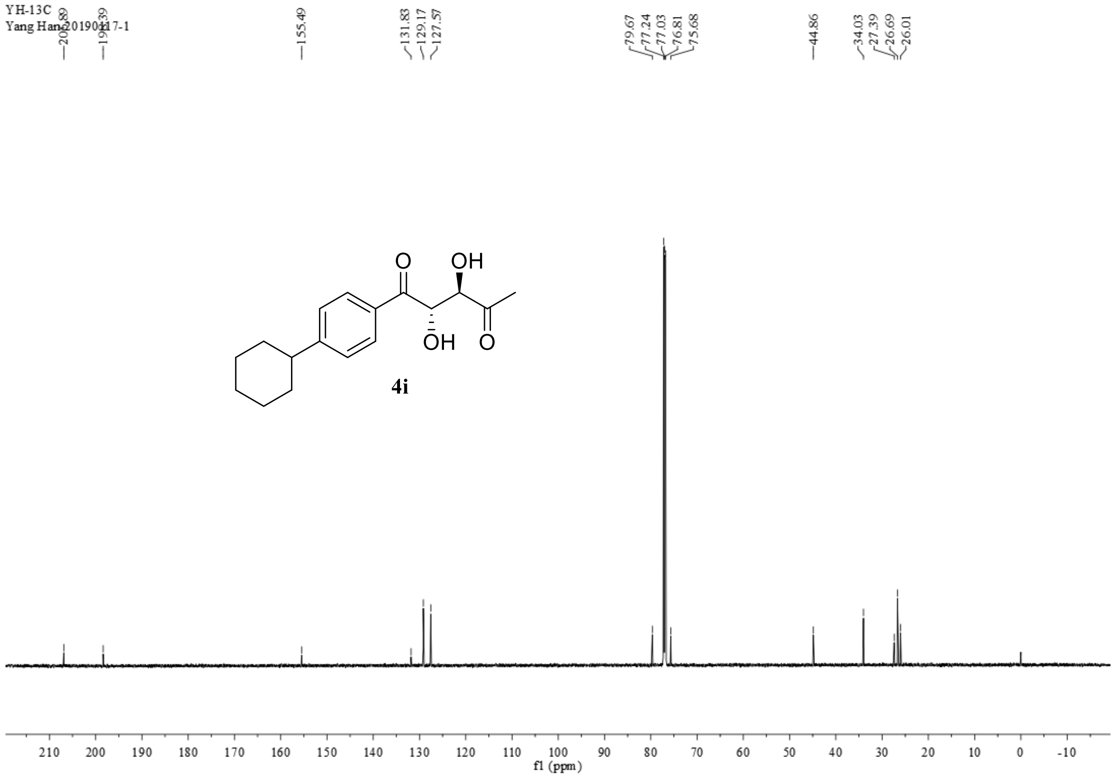
YH-13C
155.49
131.83
129.17
127.57
79.67
77.24
77.03
76.81
75.68
44.86
34.03
27.39
26.69
26.01
O
OH
OH
O
4i
f1 (ppm)

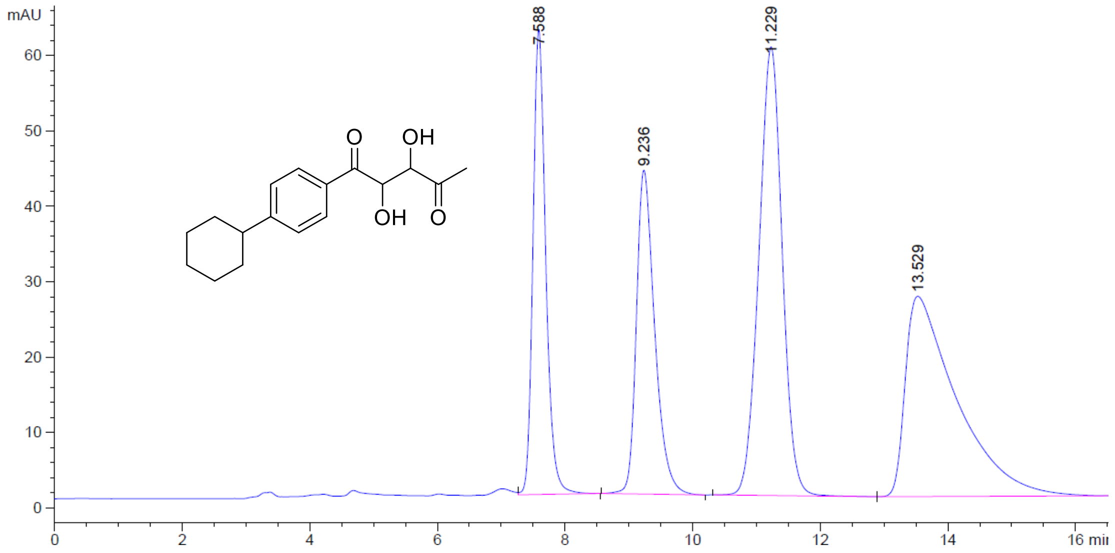

Peak #	RetTime [min]	Type	Width [min]	Area [mAU*s]	Height [mAU]	Area %
1	7.588	VB	0.2120	859.44495	61.69501	18.2287
2	9.236	BB	0.3012	857.11310	42.95889	18.1792
3	11.229	BB	0.3868	1512.38696	59.49935	32.0775
4	13.529	BB	0.7897	1485.84473	26.55414	31.5145

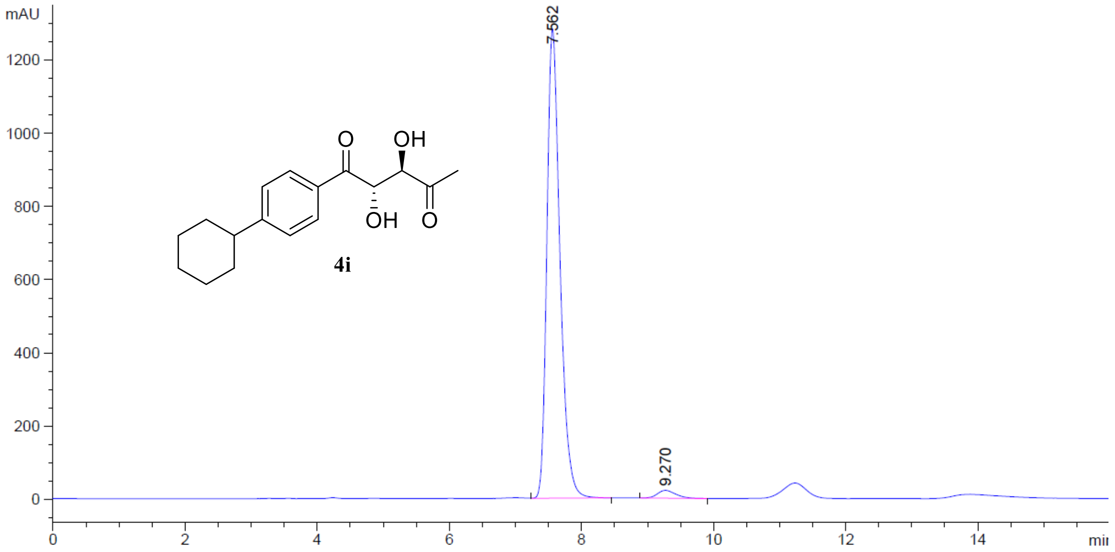

Peak #	RetTime [min]	Type	Width [min]	Area [mAU*s]	Height [mAU]	Area %
1	7.562	VB	0.2088	1.76694e4	1282.71814	97.7472
2	9.270	BB	0.2942	407.23624	21.18026	2.2528

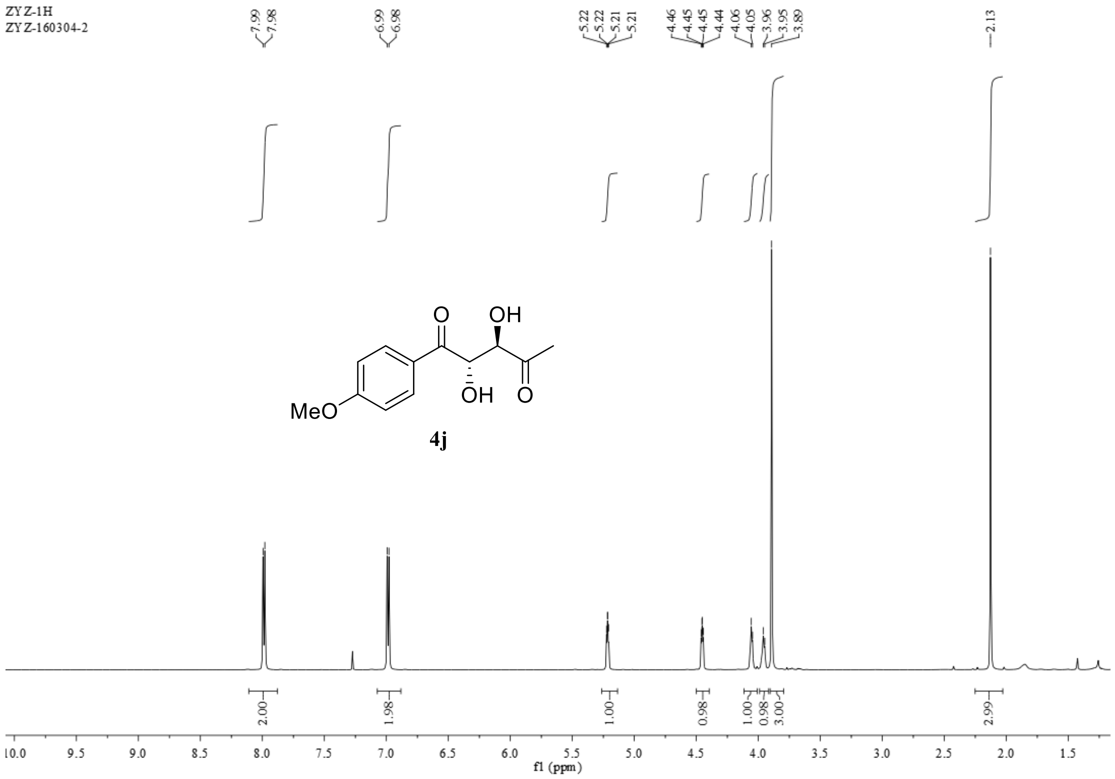
ZYZ-1H
ZYZ-160304-2
7.99
7.98
6.99
6.98
5.22
5.22
5.21
5.21
4.46
4.45
4.45
4.44
4.06
4.05
3.96
3.95
3.89
2.13
O
OH
MeO
OH
O
4j
2.00
1.98
1.00
0.98
1.00
0.98
3.00
2.99
f1 (ppm)

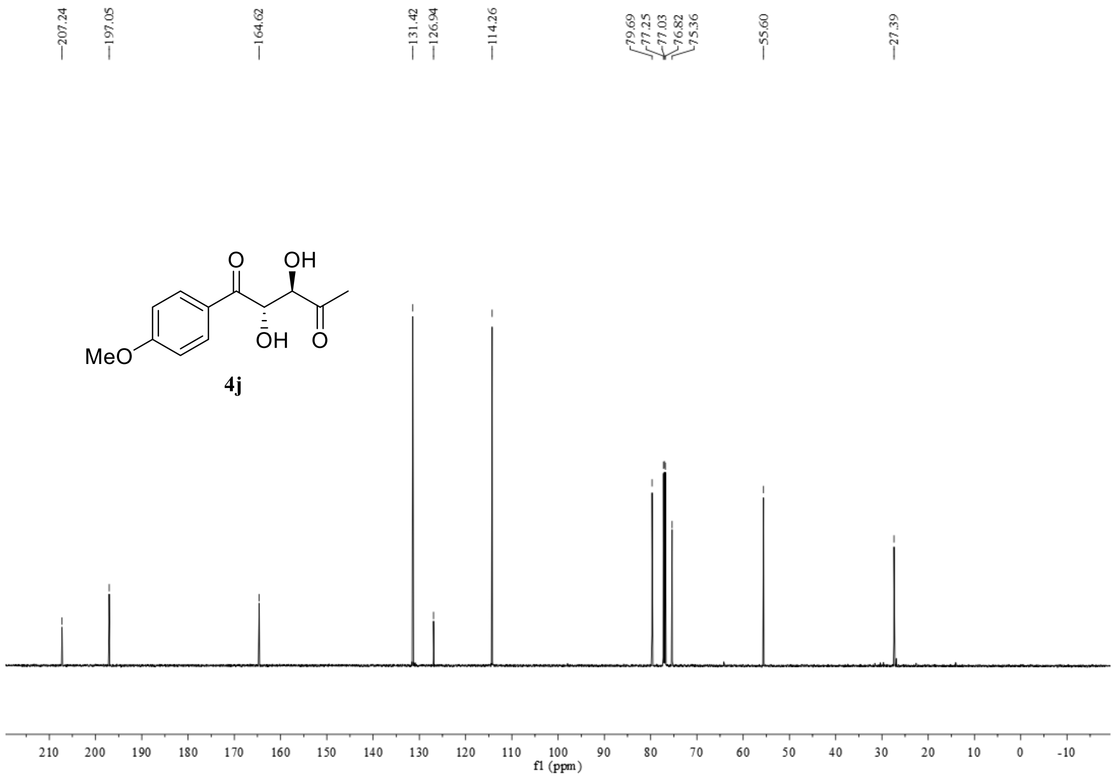
207.24
197.05
164.62
131.42
126.94
114.26
79.69
77.25
77.03
76.82
75.36
55.60
27.39
O
OH
MeO
OH
O
4j
f1 (ppm)

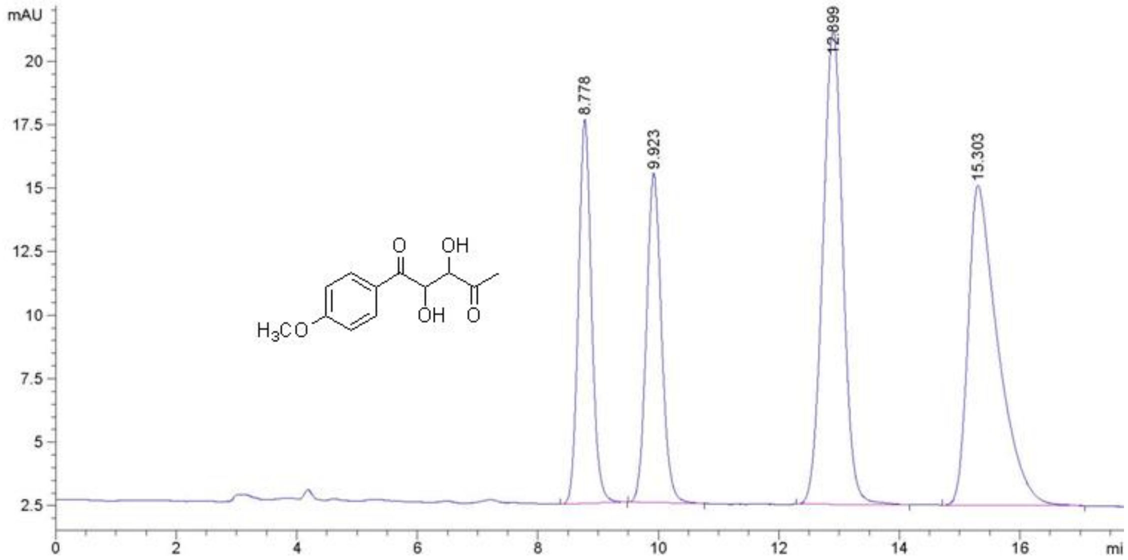

Peak #	RetTime [min]	Type	Width [min]	Area [mAU*s]	Height [mAU]	Area %
1	8.778	BB	0.2314	228.04756	15.11089	17.3210
2	9.923	BB	0.2668	225.25436	12.95990	17.1088
3	12.899	BB	0.3583	431.55206	18.68399	32.7778
4	15.303	BB	0.4917	431.74481	12.59380	32.7924

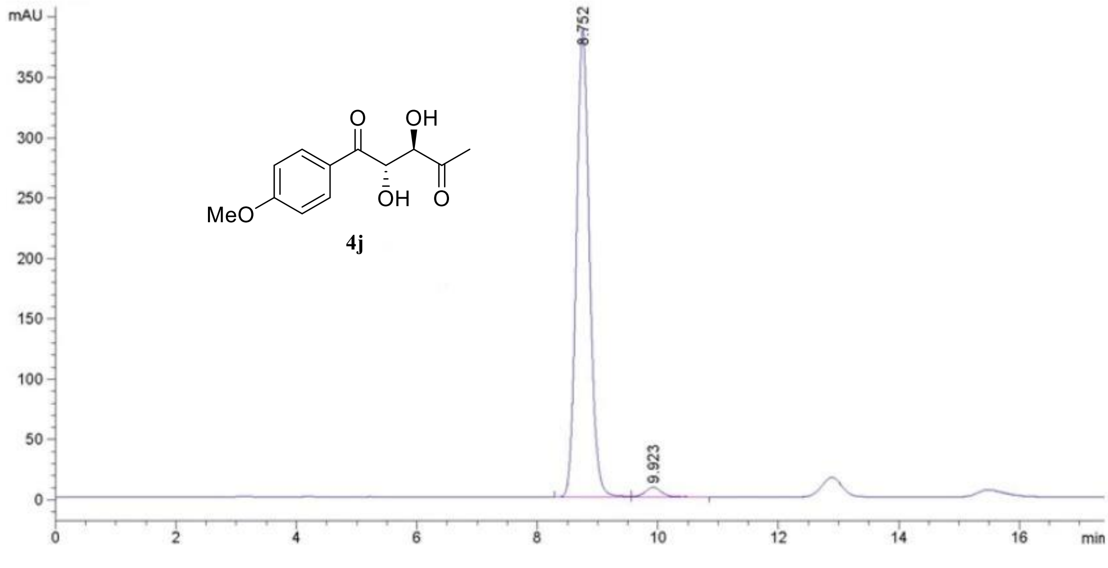

Peak #	RetTime [min]	Type	Width [min]	Area [mAU*s]	Height [mAU]	Area %
1	8.752	BV	0.2338	5869.73291	386.87262	97.6031
2	9.923	VB	0.2814	144.14372	7.78839	2.3969

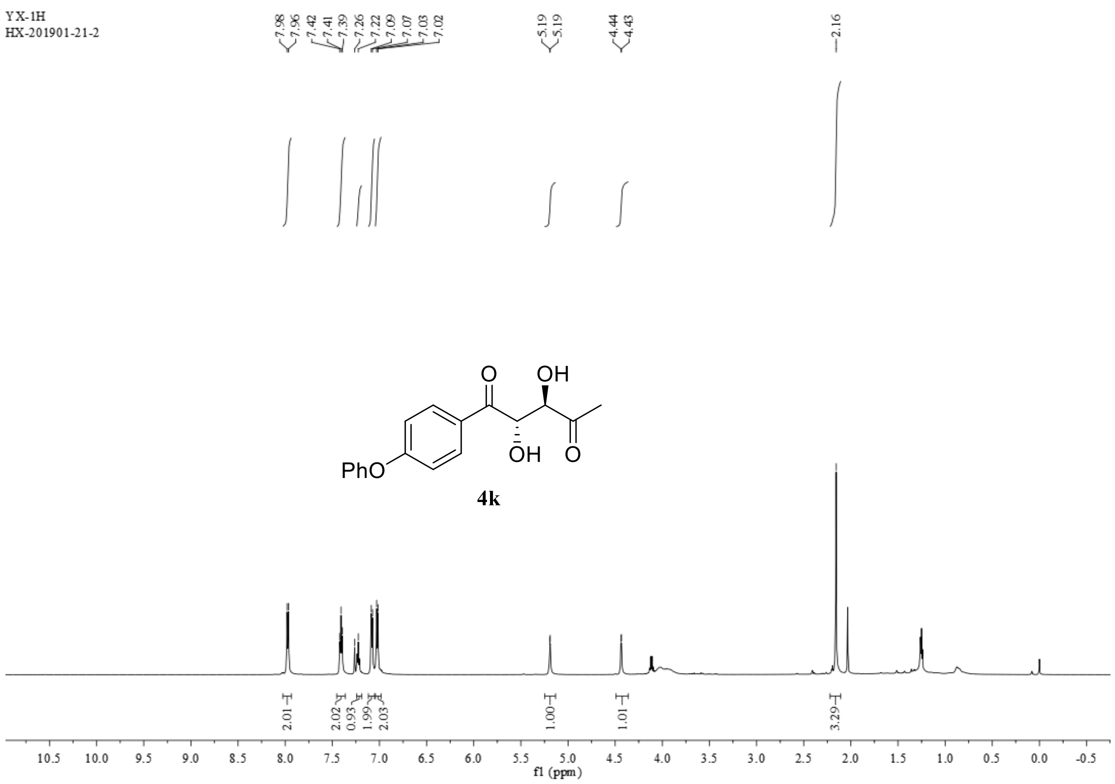
YX-1H
HX-201901-21-2
7.98 7.96 7.42 7.41 7.39 7.26 7.22 7.09 7.07 7.03 7.02
5.19 5.19
4.44 4.43
2.16
PhO
O
OH
OH
O
4k
2.01 2.02 0.93 1.99 2.03 1.00 1.01 3.29
f1 (ppm)

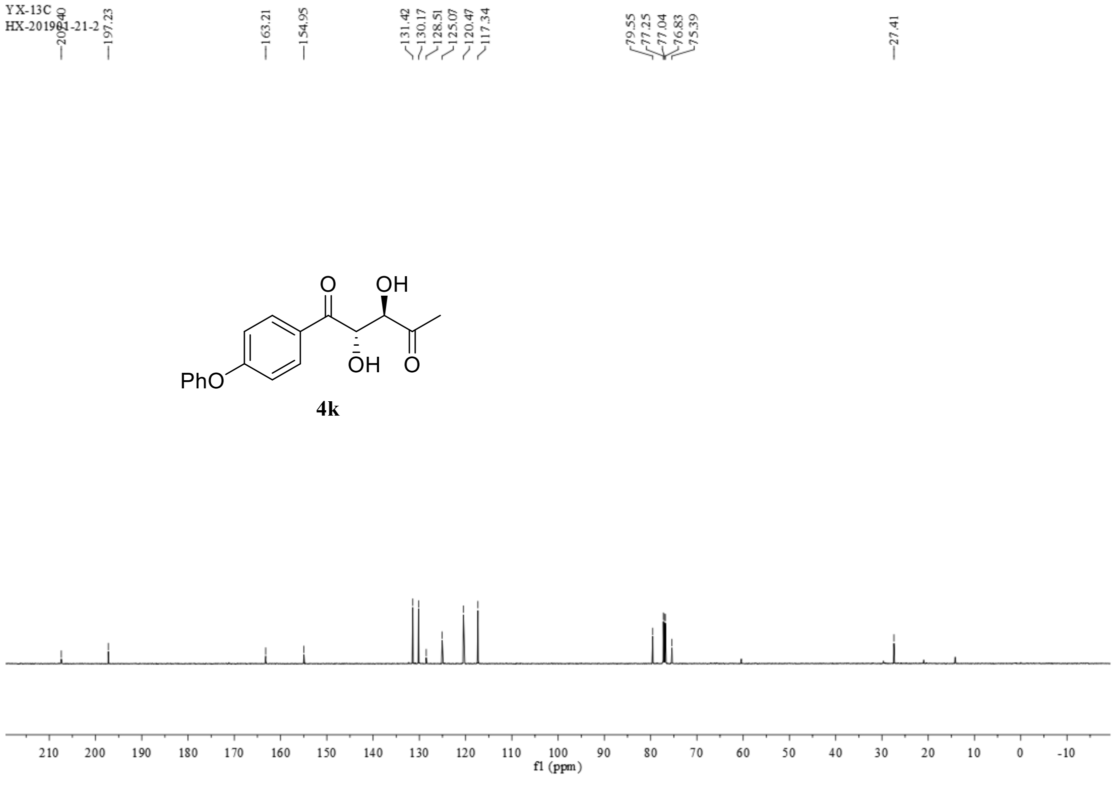
YX-13C
HX-201901-21-2
197.23
163.21
154.95
131.42 130.17 128.51 125.07 120.47 117.34
79.55 77.25 77.04 76.83 75.39
27.41
PhO
O
OH
OH
O
4k
f1 (ppm)

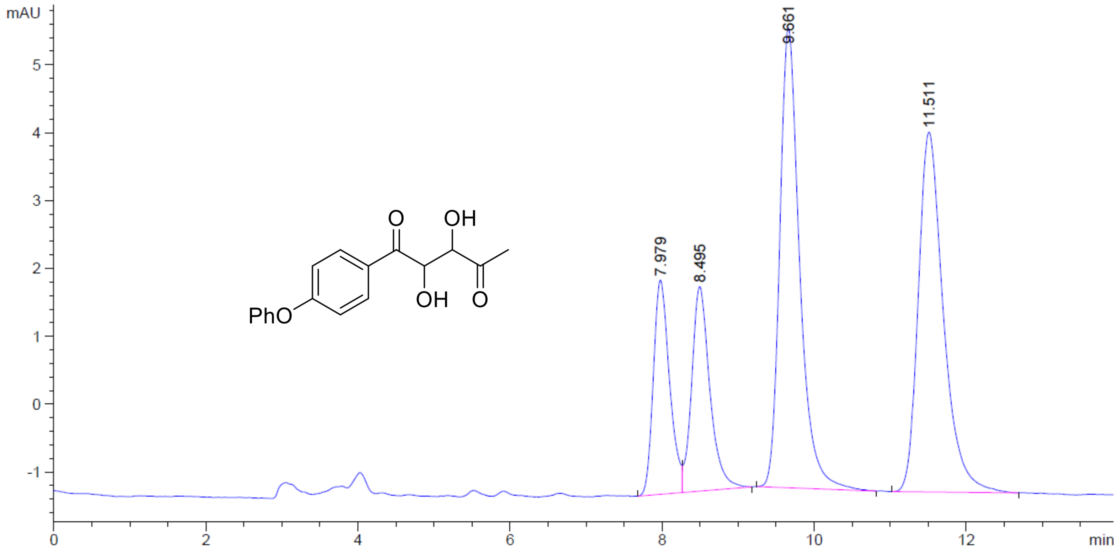

Peak #	RetTime [min]	Type	Width [min]	Area [mAU*s]	Height [mAU]	Area %
1	7.979	BV	0.2249	46.66187	3.15618	13.6567
2	8.495	VB	0.2490	49.99683	3.01252	14.6328
3	9.661	BB	0.2735	123.27216	6.77283	36.0786
4	11.511	BB	0.3451	121.74620	5.30158	35.6320

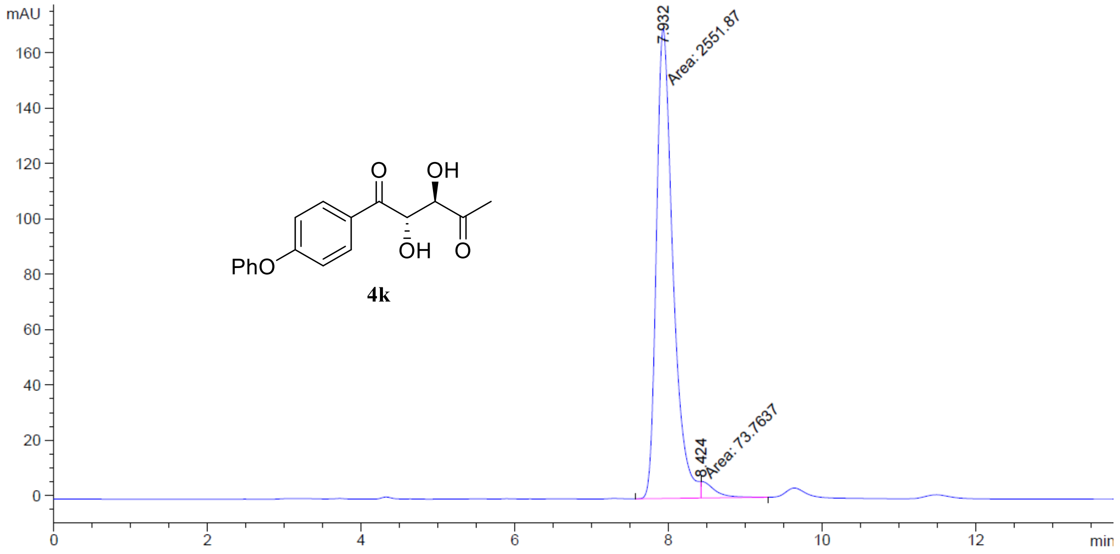

Peak #	RetTime [min]	Type	Width [min]	Area [mAU*s]	Height [mAU]	Area %
1	7.932	MF	0.2504	2551.86865	169.84280	97.1906
2	8.424	FM	0.2048	73.76369	6.00317	2.8094

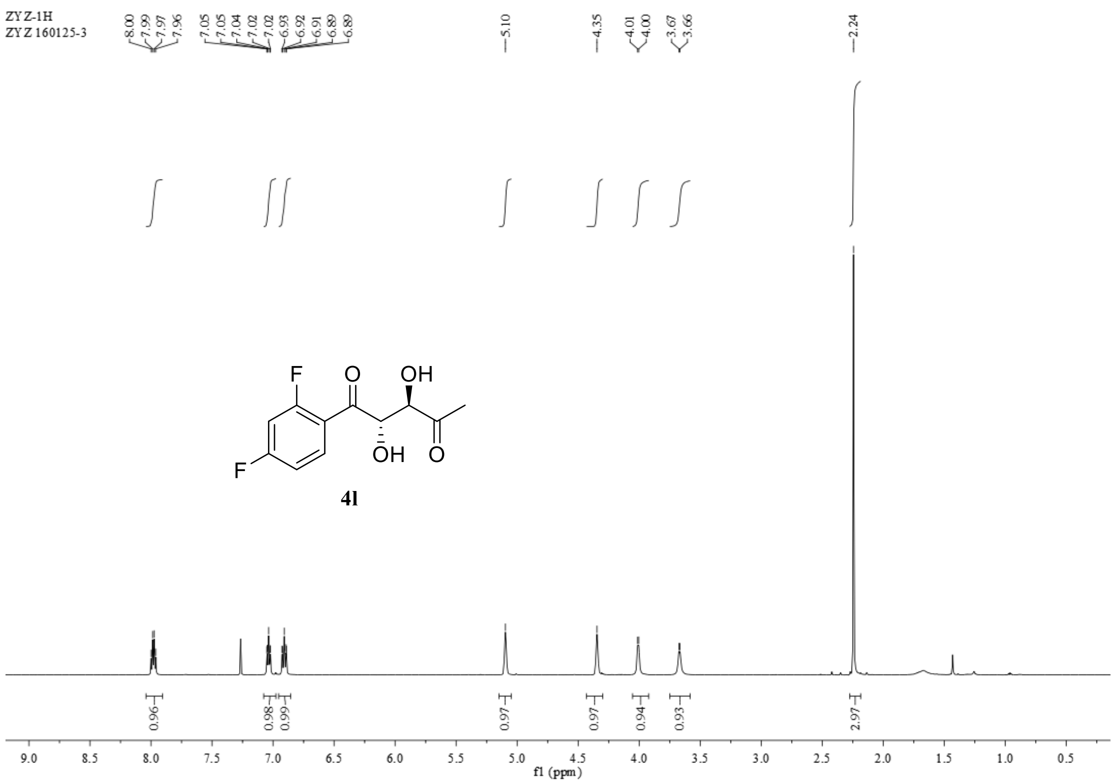
ZYZ-1H
ZYZ 160125-3
8.00
7.99
7.97
7.96
7.05
7.05
7.04
7.02
7.02
6.93
6.92
6.91
6.89
6.89
5.10
4.35
4.01
4.00
3.67
3.66
2.24
F
O
OH
OH
O
F
4l
0.96
0.98
0.99
0.97
0.97
0.94
0.93
2.97
f1 (ppm)

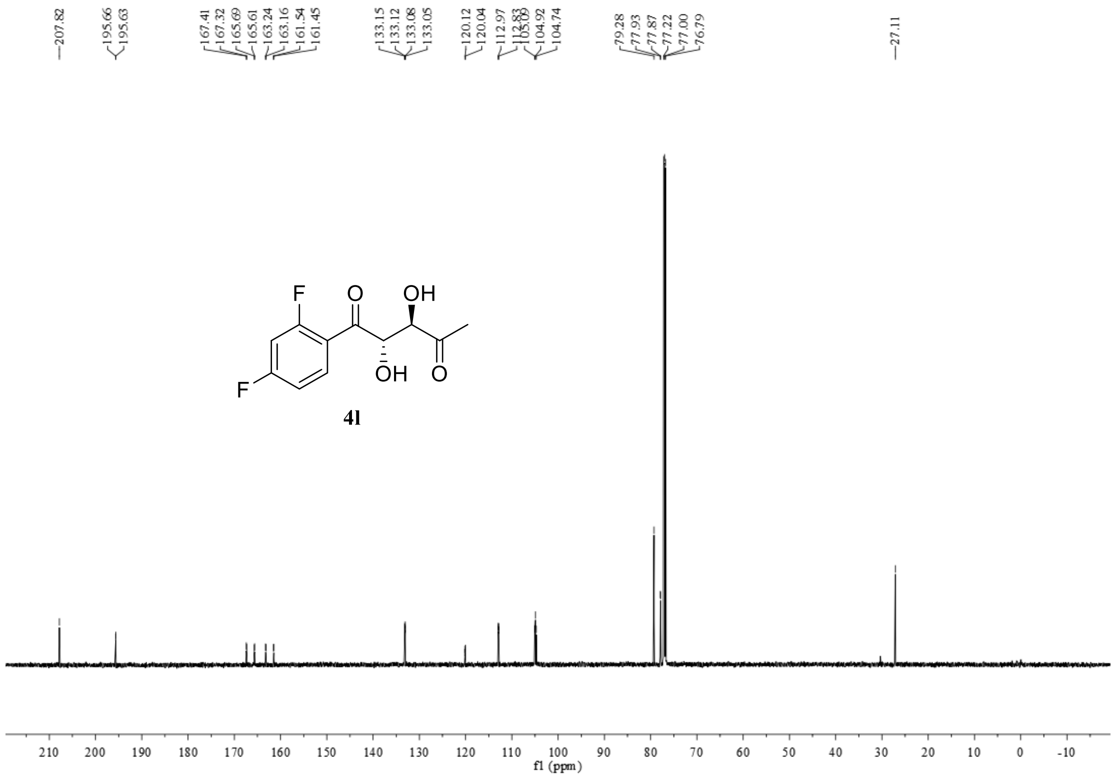
207.82
195.66
195.63
167.41
167.32
165.69
165.61
163.24
163.16
161.54
161.45
133.15
133.12
133.08
133.05
120.12
120.04
112.97
112.83
104.92
104.74
79.28
77.93
77.87
77.22
77.00
76.79
27.11
F
O
OH
OH
O
F
4l
f1 (ppm)

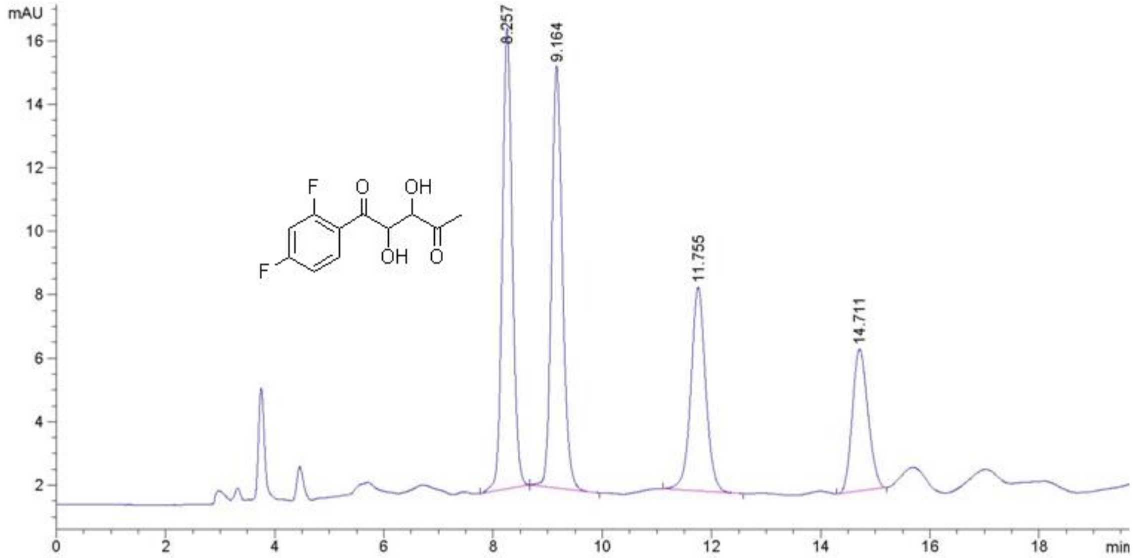

Peak #	RetTime [min]	Type	Width [min]	Area [mAU*s]	Height [mAU]	Area %
1	8.257	BB	0.1963	184.36545	14.50908	31.9382
2	9.164	BB	0.2131	184.67561	13.28911	31.9920
3	11.755	BB	0.2823	119.87582	6.40900	20.7665
4	14.711	BB	0.3081	88.33936	4.46305	15.3033

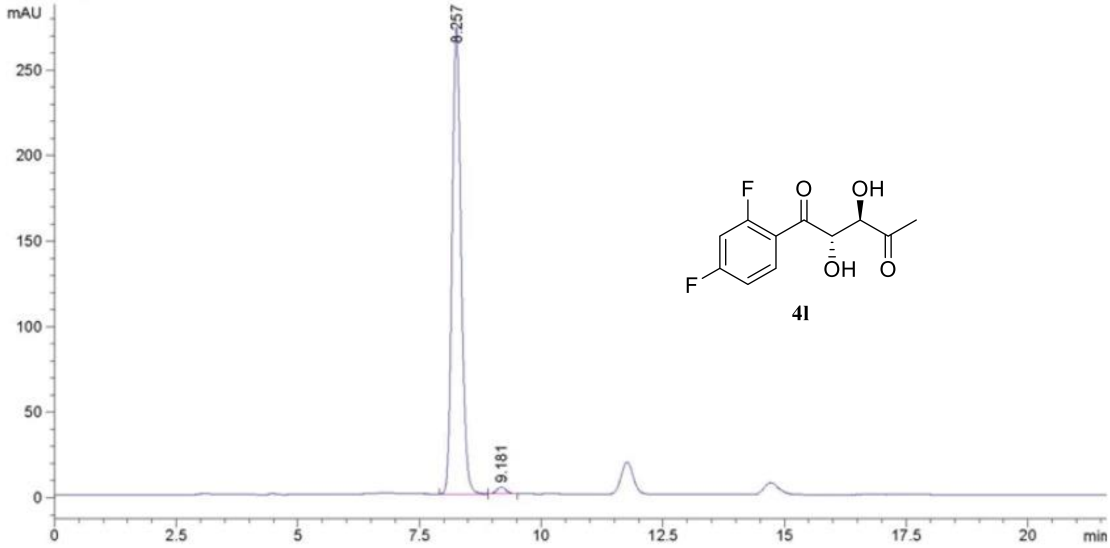

Peak #	RetTime [min]	Type	Width [min]	Area [mAU*s]	Height [mAU]	Area %
1	8.257	BV	0.1977	3496.92529	272.58347	98.5221
2	9.181	VB	0.2112	52.45619	3.81888	1.4779

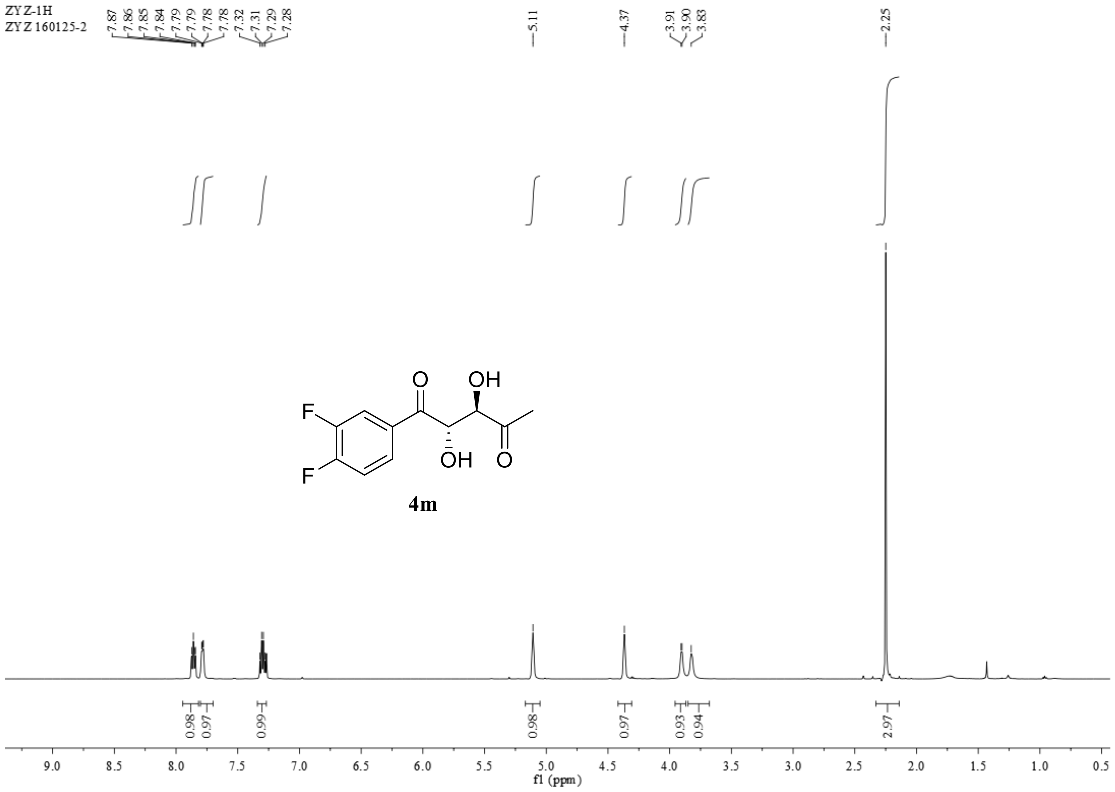
ZYZ-1H
ZYZ 160125-2
7.87 7.86 7.85 7.84 7.79 7.79 7.78 7.78 7.32 7.31 7.29 7.28
5.11
4.37
3.91 3.90 3.83
2.25
F
F
O
OH
OH
O
4m
0.98 0.97 0.99 0.98 0.97 0.93 0.94 2.97
9.0 8.5 8.0 7.5 7.0 6.5 6.0 5.5 5.0 4.5 4.0 3.5 3.0 2.5 2.0 1.5 1.0 0.5
f1 (ppm)

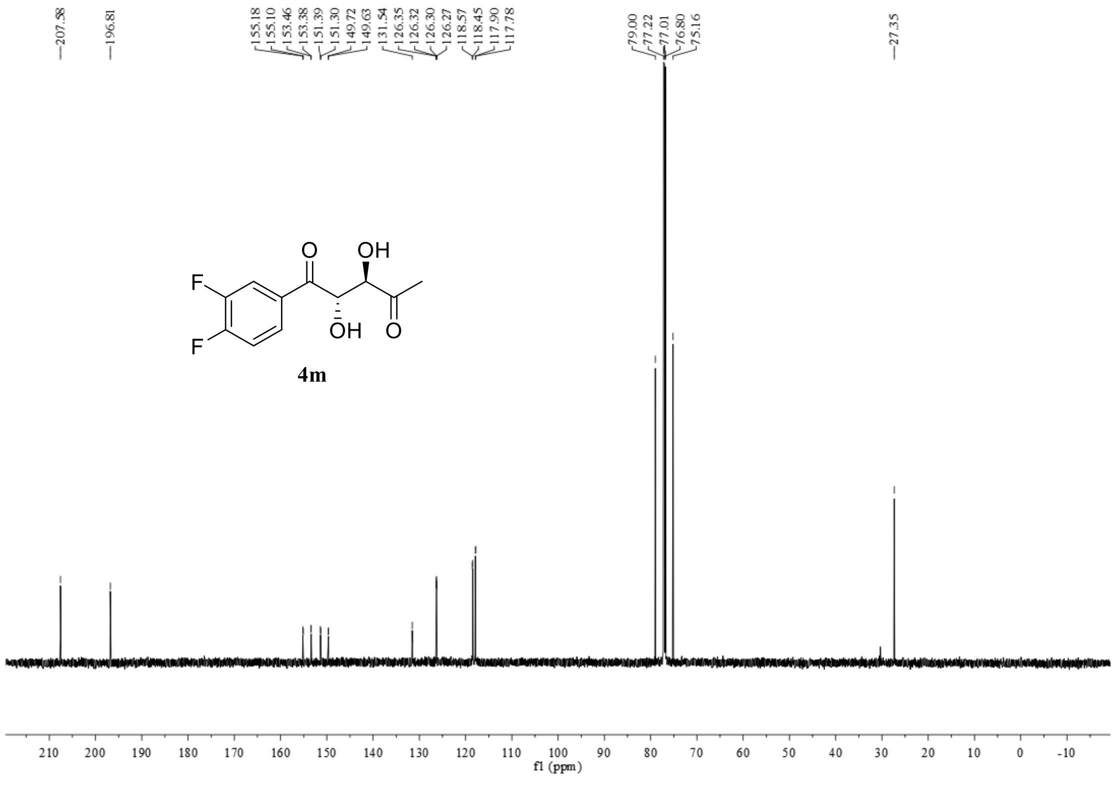
207.58
196.81
155.18 155.10 153.46 153.38 151.39 151.30 149.72 149.63 131.54 126.35 126.32 126.30 126.27 118.57 118.45 117.90 117.78
79.00 77.22 77.01 76.80 75.16
27.35
F
F
O
OH
OH
O
4m
210 200 190 180 170 160 150 140 130 120 110 100 90 80 70 60 50 40 30 20 10 0 -10
f1 (ppm)

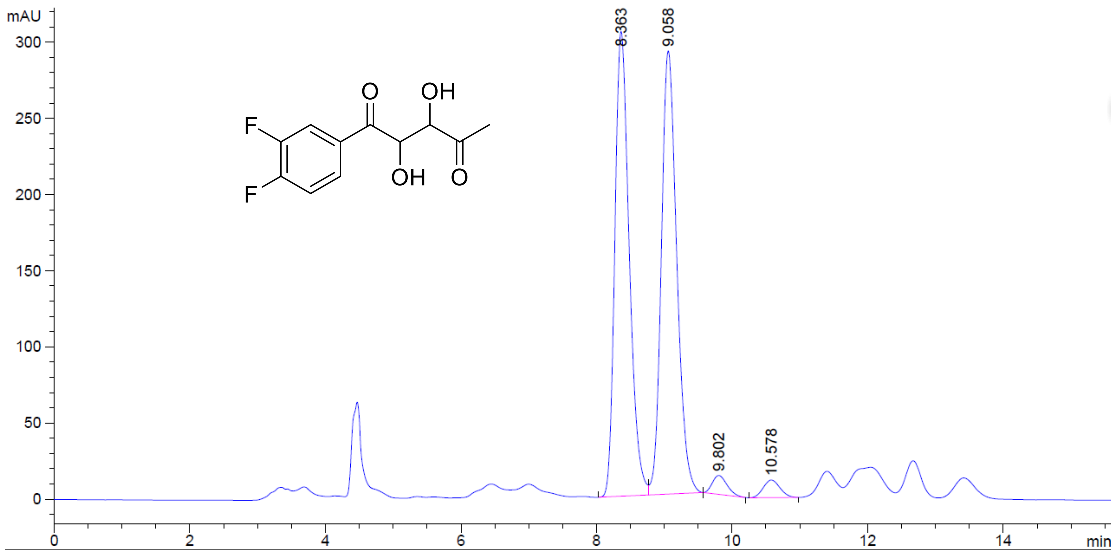

Peak #	RetTime [min]	Type	Width [min]	Area [mAU*s]	Height [mAU]	Area %
1	8.363	BV	0.2259	4499.51660	305.18317	47.4124
2	9.058	VB	0.2430	4606.69238	291.04071	48.5418
3	9.802	BB	0.2355	186.94438	12.41215	1.9699
4	10.578	BB	0.2663	197.00714	11.53703	2.0759

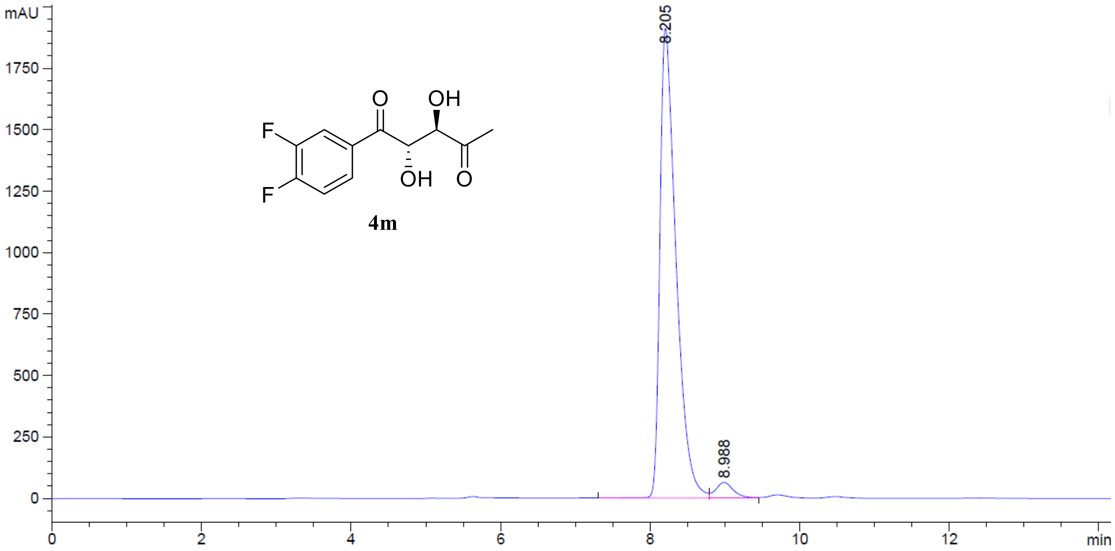

Peak #	RetTime [min]	Type	Width [min]	Area [mAU*s]	Height [mAU]	Area %
1	8.205	BV	0.2241	2.85949e4	1911.06152	96.4034
2	8.988	VV	0.2481	1066.81079	64.09940	3.5966

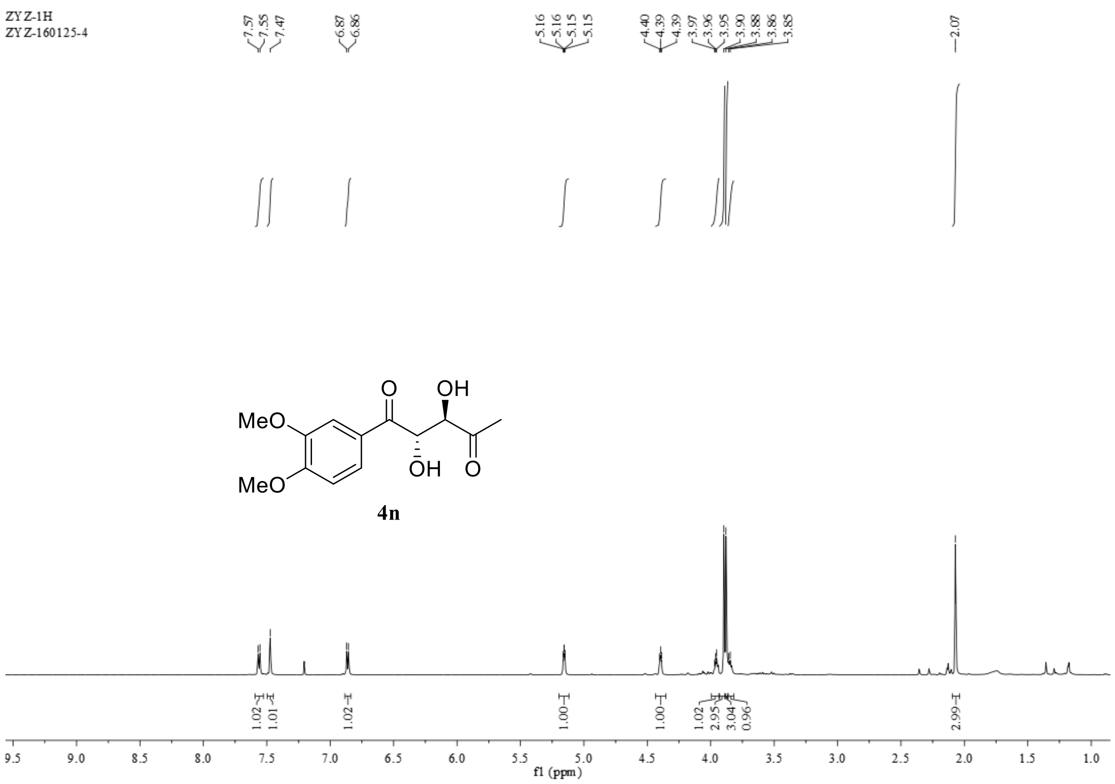
ZYZ-1H
ZYZ-160125-4
7.57
7.55
7.47
6.87
6.86
5.16
5.16
5.15
5.15
4.40
4.39
4.39
3.97
3.96
3.95
3.90
3.88
3.86
3.85
2.07
MeO
MeO
O
OH
OH
O
4n
1.02
1.01
1.02
1.00
1.00
1.02
2.95
3.04
0.96
2.99
f1 (ppm)

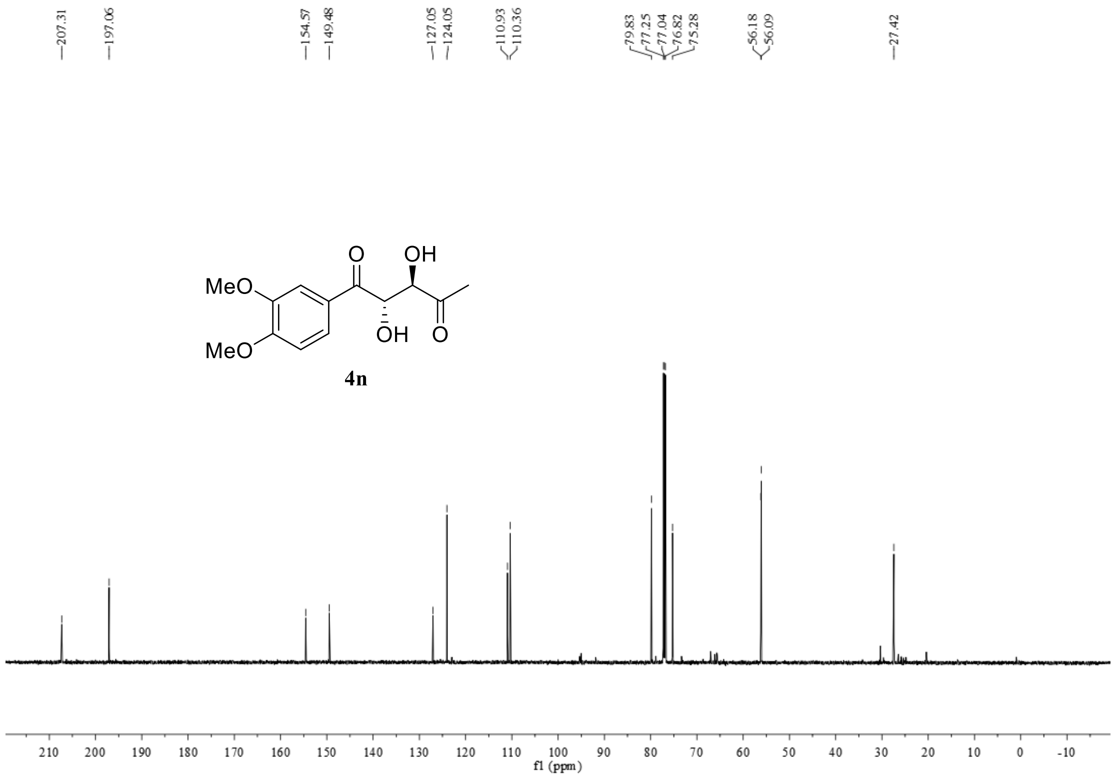
207.31
197.06
154.57
149.48
127.05
124.05
110.93
110.36
79.83
77.25
77.04
76.82
75.28
56.18
56.09
27.42
MeO
MeO
O
OH
OH
O
4n
f1 (ppm)

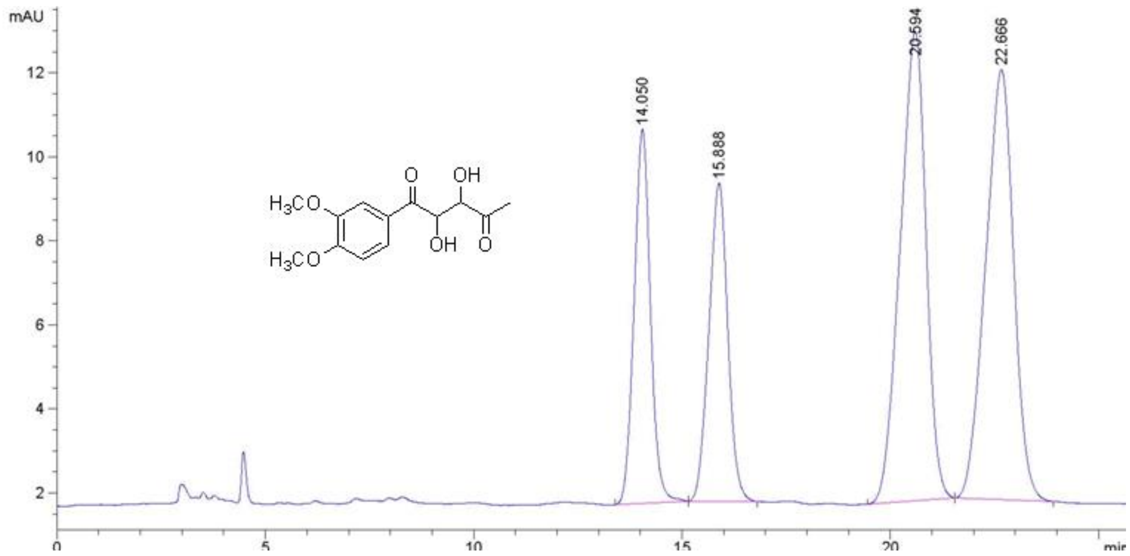

Peak #	RetTime [min]	Type	Width [min]	Area [mAU*s]	Height [mAU]	Area %
1	14.050	BB	0.4112	241.49118	8.89995	16.7691
2	15.888	BB	0.4780	237.38399	7.56985	16.4839
3	20.594	BB	0.6405	481.05862	11.17663	33.4046
4	22.666	BB	0.6854	480.16418	10.23913	33.3425

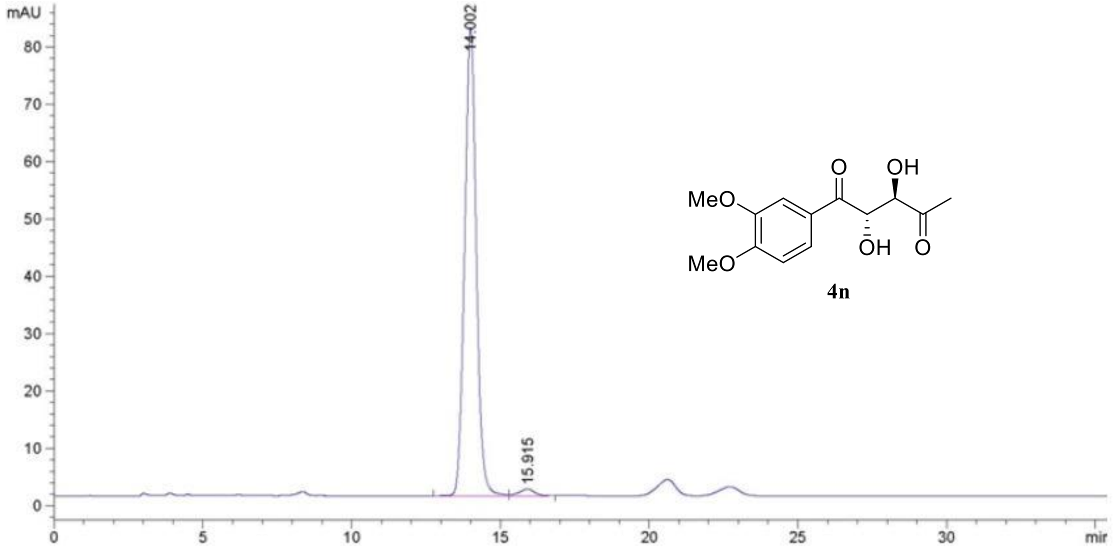

Peak #	RetTime [min]	Type	Width [min]	Area [mAU*s]	Height [mAU]	Area %
1	14.002	BV	0.4137	2223.37061	81.67034	98.1808
2	15.915	VB	0.5097	41.19763	1.18730	1.8192

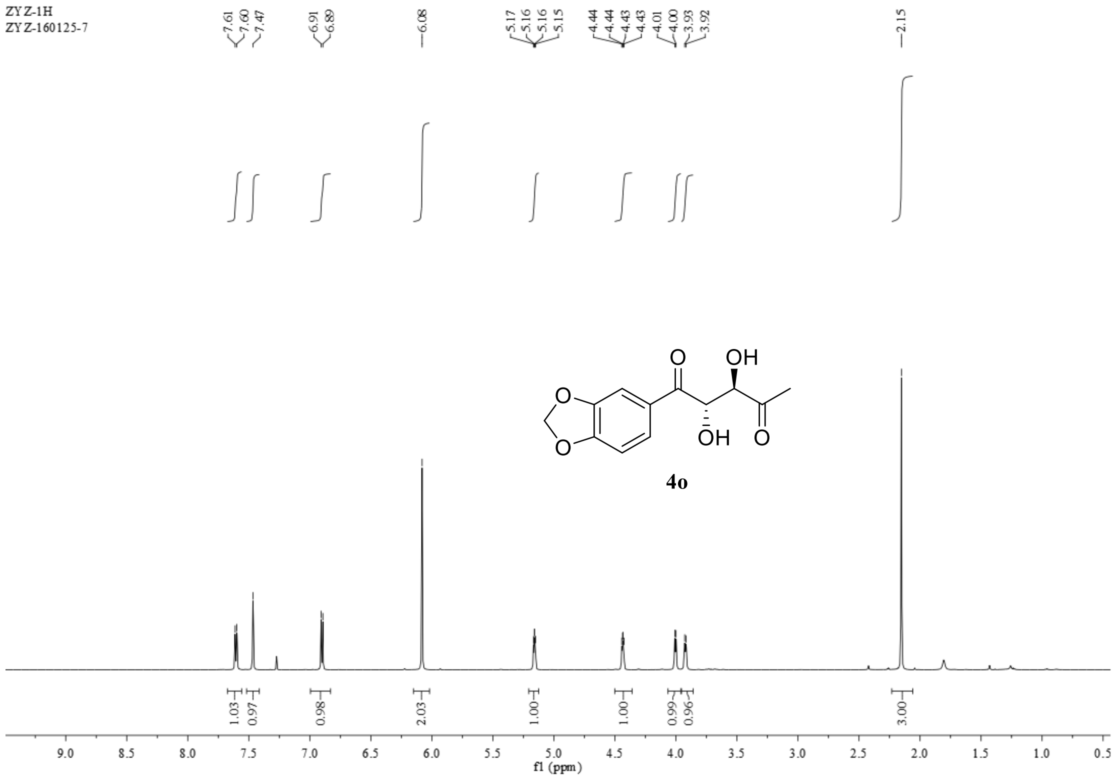
ZYZ-1H
ZYZ-160125-7
7.61
7.60
7.47
6.91
6.89
6.08
5.17
5.16
5.16
5.15
4.44
4.44
4.43
4.43
4.01
4.00
3.93
3.92
2.15
O
OH
O
O
OH
O
4o
1.03
0.97
0.98
2.03
1.00
1.00
0.99
0.96
3.00
f1 (ppm)

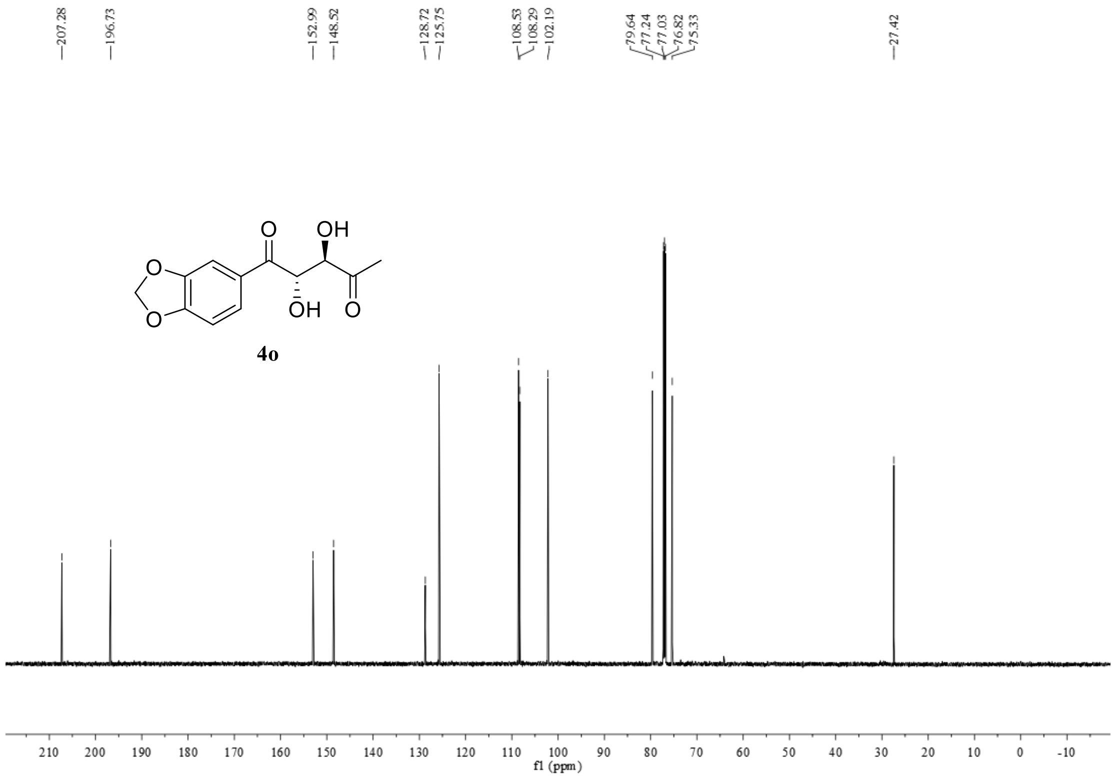
207.28
196.73
152.99
148.52
128.72
125.75
108.53
108.29
102.19
79.64
77.24
77.03
76.82
75.33
27.42
O
OH
O
O
OH
O
4o
f1 (ppm)

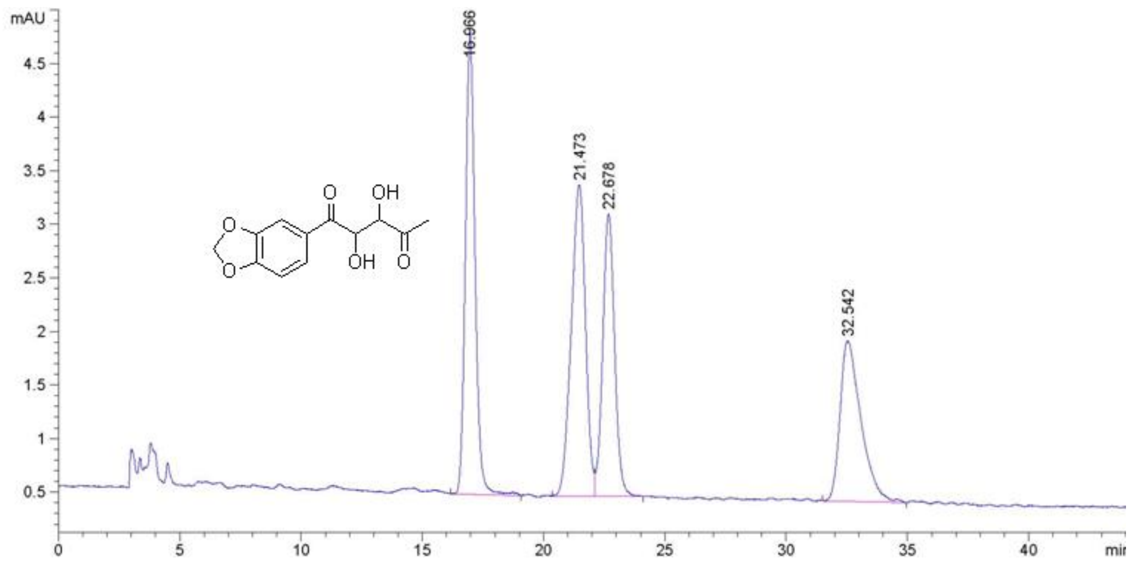

Peak #	RetTime [min]	Type	Width [min]	Area [mAU*s]	Height [mAU]	Area %
1	16.966	BB	0.4310	120.22090	4.29997	28.7024
2	21.473	BV	0.6233	119.03335	2.90692	28.4189
3	22.678	VB	0.5309	89.72453	2.63686	21.4215
4	32.542	BB	0.8688	89.87440	1.49802	21.4573

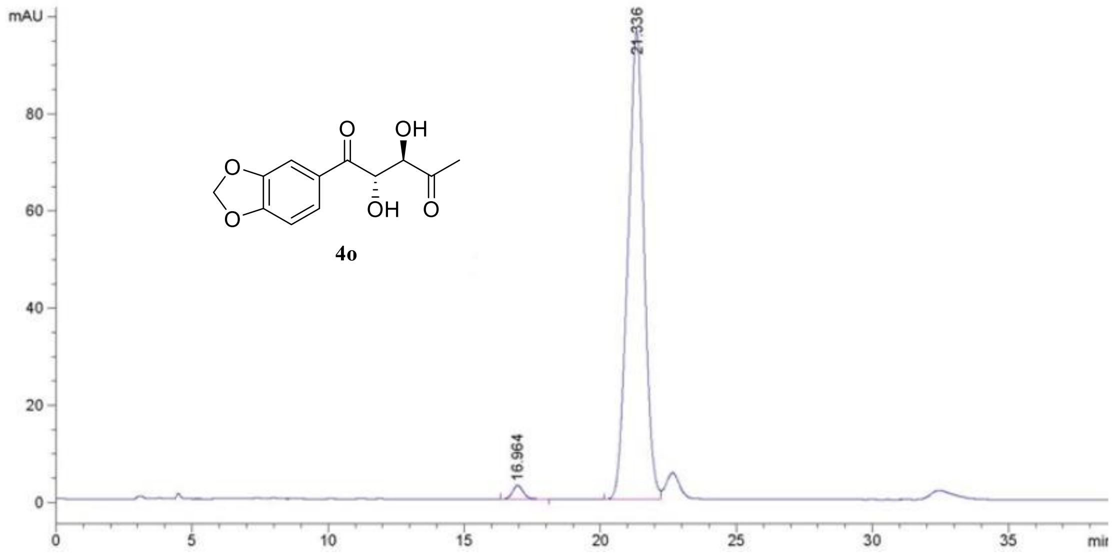

Peak #	RetTime [min]	Type	Width [min]	Area [mAU*s]	Height [mAU]	Area %
1	16.964	BB	0.4121	76.99448	2.81670	1.9011
2	21.336	BV	0.6104	3972.93457	96.39190	98.0989

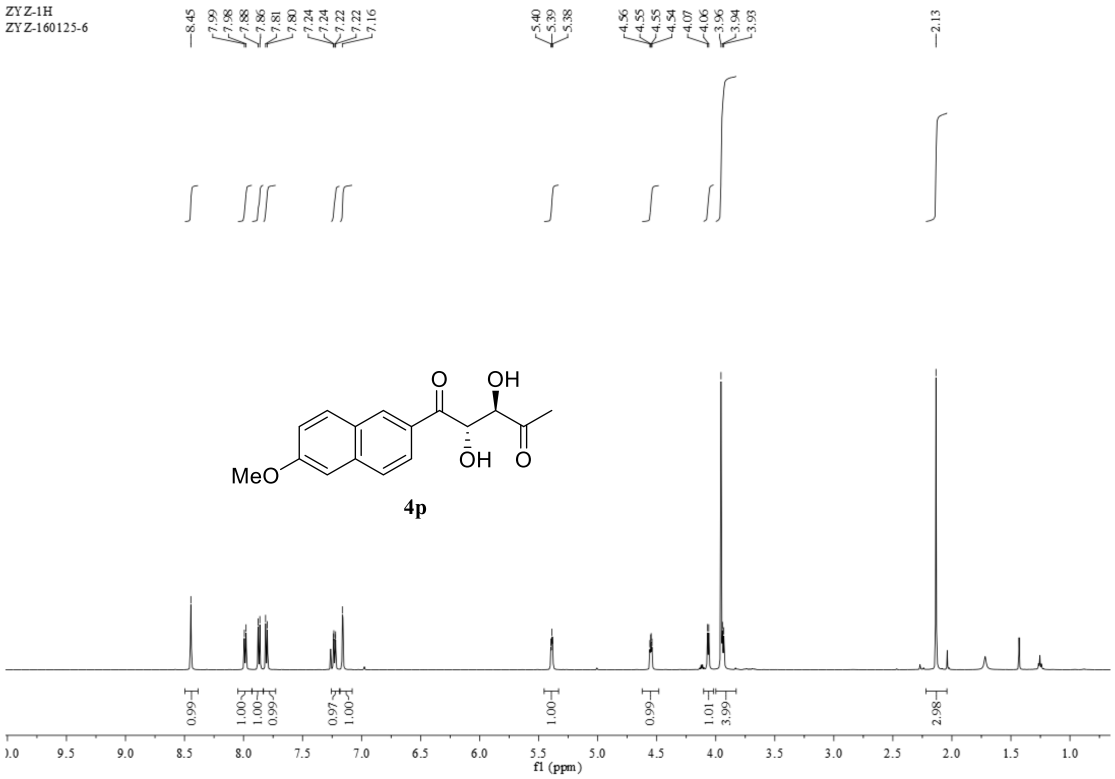
ZYZ-1H
ZYZ-160125-6
8.45
7.99
7.98
7.88
7.86
7.81
7.80
7.24
7.24
7.22
7.22
7.16
5.40
5.39
5.38
4.56
4.55
4.55
4.54
4.07
4.06
3.96
3.94
3.93
2.13
MeO
O
OH
OH
O
4p
0.99
1.00
1.00
0.99
0.97
1.00
1.00
0.99
1.01
3.99
2.98
f1 (ppm)

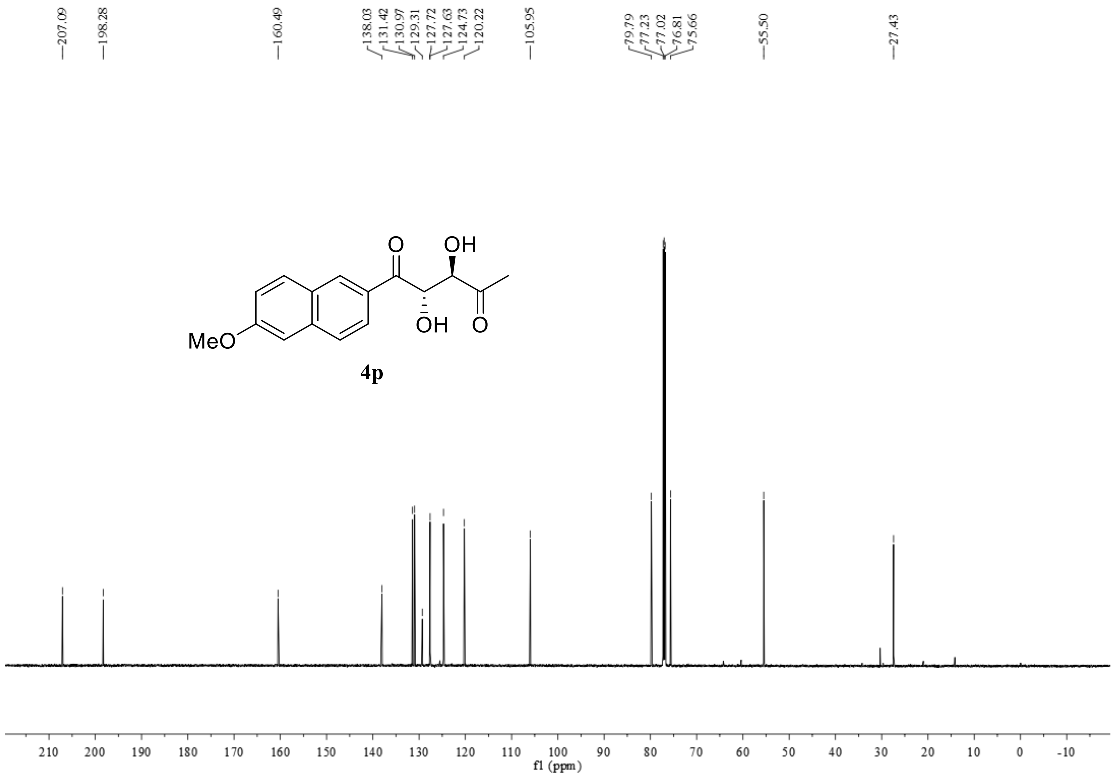
207.09
198.28
160.49
138.03
131.42
130.97
129.31
127.72
127.63
124.73
120.22
105.95
79.79
77.23
77.02
76.81
75.66
55.50
27.43
MeO
O
OH
OH
O
4p
f1 (ppm)

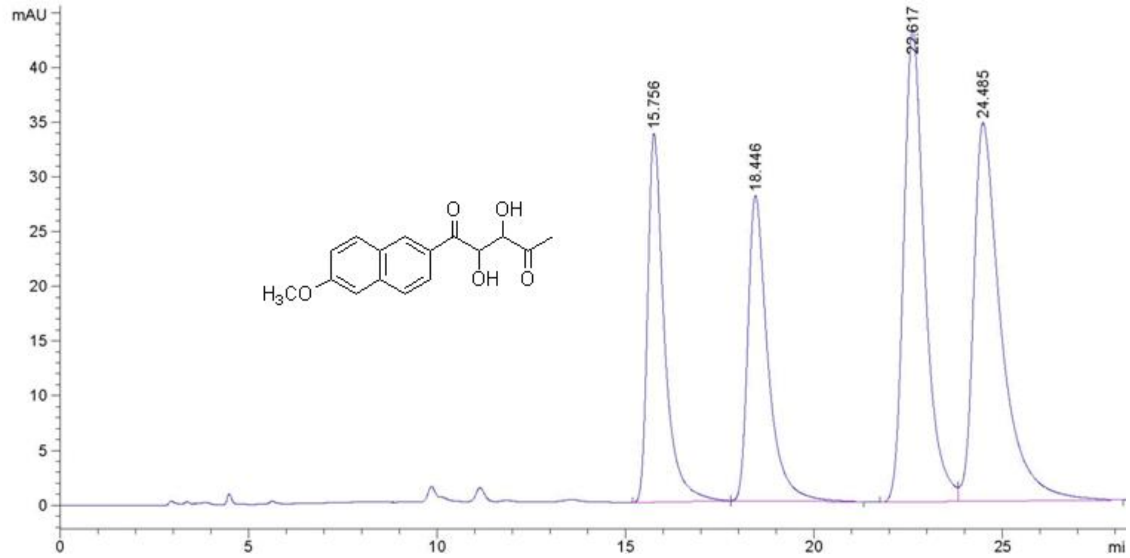

Peak #	RetTime [min]	Type	Width [min]	Area [mAU*s]	Height [mAU]	Area %
1	15.756	BB	0.4560	1035.28479	33.69727	18.8339
2	18.446	BB	0.5452	1025.22119	27.90237	18.6509
3	22.617	BV	0.5869	1679.81372	43.12879	30.5592
4	24.485	VB	0.7423	1756.59265	34.59644	31.9560

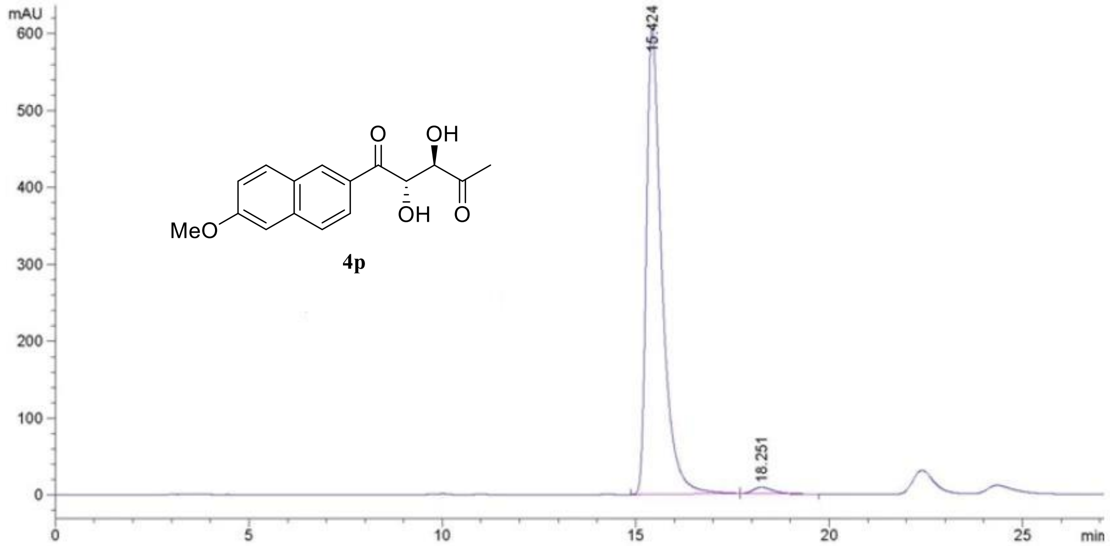

Peak #	RetTime [min]	Type	Width [min]	Area [mAU*s]	Height [mAU]	Area %
1	15.424	BB	0.4154	1.68834e4	605.76044	98.3467
2	18.251	BB	0.5174	283.83081	8.20088	1.6533
